# Head anatomy and phylogenomics show the Carboniferous giant *Arthropleura* belonged to a millipede-centipede group

**DOI:** 10.1126/sciadv.adp6362

**Published:** 2024-10-09

**Authors:** Mickaël Lhéritier, Gregory D. Edgecombe, Russell J. Garwood, Adrien Buisson, Alexis Gerbe, Nicolás Mongiardino Koch, Jean Vannier, Gilles Escarguel, Jérome Adrien, Vincent Fernandez, Aude Bergeret-Medina, Vincent Perrier

**Affiliations:** ^1^ Universite Claude Bernard Lyon 1, CNRS, ENS de Lyon, LGL-TPE UMR 5276, F-69622 Villeurbanne.; ^2^ Universite Claude Bernard Lyon 1, LEHNA UMR 5023, CNRS, ENTPE, F-69622, Villeurbanne, France.; ^3^ The Natural History Museum, London SW7 5BD, UK.; ^4^ Department of Earth and Environmental Sciences, University of Manchester, Manchester M13 9PL, UK.; ^5^ Scripps Institution of Oceanography, University of California San Diego, La Jolla, CA, USA.; ^6^ Laboratoire MATEIS. INSA Lyon, Jules Verne building, 21, avenue Jean Capelle, 69621 Villeurbanne Cedex, France.; ^7^ European Synchrotron Radiation Facility, 71 rue des Martyrs, 38000 Grenoble, France.; ^8^ Muséum d’Histoire Naturelle d’Autun, 14, rue Saint Antoine, 71400 Autun, France.

## Abstract

The Carboniferous myriapod *Arthropleura* is the largest arthropod of all time, but its fossils are usually incomplete, limiting the understanding of its anatomy, ecology, and relationships. Micro–computed tomography applied to exceptionally preserved specimens from the Carboniferous Montceau-les-Mines Lagerstätte (France) reveals unprecedented details of its functional anatomy, such as the head and mouthparts. *Arthropleura* shares features with both millipedes and centipedes. Total-evidence phylogeny combining morphological and transcriptomic data resolves *Arthropleura* alone as a stem group millipede, but the inclusion of the highly incomplete Siluro-Devonian *Eoarthropleura* draws it deeper into the myriapod stem. *Arthropleura* suggests transitional morphology between clades united primarily by molecular information and underscores the value of total-evidence phylogenetics to understanding evolutionary history.

## Teaser

3D data of the heads of Arthropleura show that it shares trunk affinities with millipedes and head affinities with centipedes.

## INTRODUCTION

The iconic myriapod *Arthropleura* is a Carboniferous-Permian arthropod renowned for its record-breaking gigantism (*1*), inhabiting forest environments near the equatorial belt (*2*) from ~346 million to 290 million years ago (Ma) (Visean to Sakmarian) (*1*). Despite its familiarity, there remain notable gaps in knowledge of the anatomy and lifestyle of *Arthropleura*, and its phylogenetic relationships have not been explored in light of the current understanding of arthropod phylogeny recovered from genomic-scale molecular data. It is now almost universally accepted that arthropleurideans [a Paleozoic group including *Arthropleura*, the late Silurian-Devonian *Eoarthropleura*, and the Devonian *Microdecemplex* (*3*, *4*)] are myriapods, a clade of terrestrial arthropods whose diversity today is dominated by centipedes and millipedes [Chilopoda and Diplopoda, respectively (*4*)]. While the monophyly of arthropleurideans is debated (*3*, *5*), a consensus has emerged that these Paleozoic giants are most closely related to millipedes and might even fall within their crown group (*3*, *6*).

The evolutionary position of arthropleurideans has until now been appraised with almost no information about the head of *Arthropleura.* The only known heads from the subclass Arthropleuridea come from a few specimens of *Microdecemplex* (*3*), partial heads of *Arthropleura* in fragmented specimens (*5*), and a partial mandible of *Eoarthropleura* (*4*, *5*). A plate interpreted as the head in many studies of *Arthropleura* was revealed to be instead the collum, variably regarded as a tergite receiving contributions from the head and trunk (*7*) or representing the first postcephalic tergite (*5*). The true head is largely covered by the collum (*8*). Nevertheless, fundamental characteristics of the head—such as the antennae (apart from possible sockets) (*5*), eyes, and many details of the mouthparts—remain unknown, limiting a robust assessment of the group’s affinities. Here, x-ray micro–computed tomography (both standard and propagation phase contrast synchrotron μCT) of juvenile, three-dimensionally preserved specimens in sideritic nodules from the Upper Carboniferous Montceau-les-Mines Lagerstätte (Kasimovian, ~305 Ma) (figs. S1 and S3) (*9*, *10*) lifts the veil on the head of *Arthropleura*. It reveals unprecedented details of its mouthparts and provides data for testing the systematic position of arthropleurideans.

## RESULTS

The Montceau specimens show clear characteristics of the order Arthropleurida, such as the presence of irregular large and small tubercles on trilobed dorsal tergites (*11*, *12*), and details of the head, which we outline below. The studied specimens are shown in thefigures. Measurements are recorded in tables S1 to S8, and details of the systematics are present in Supplementary Text.

### Preservation

**Fig. 1.**
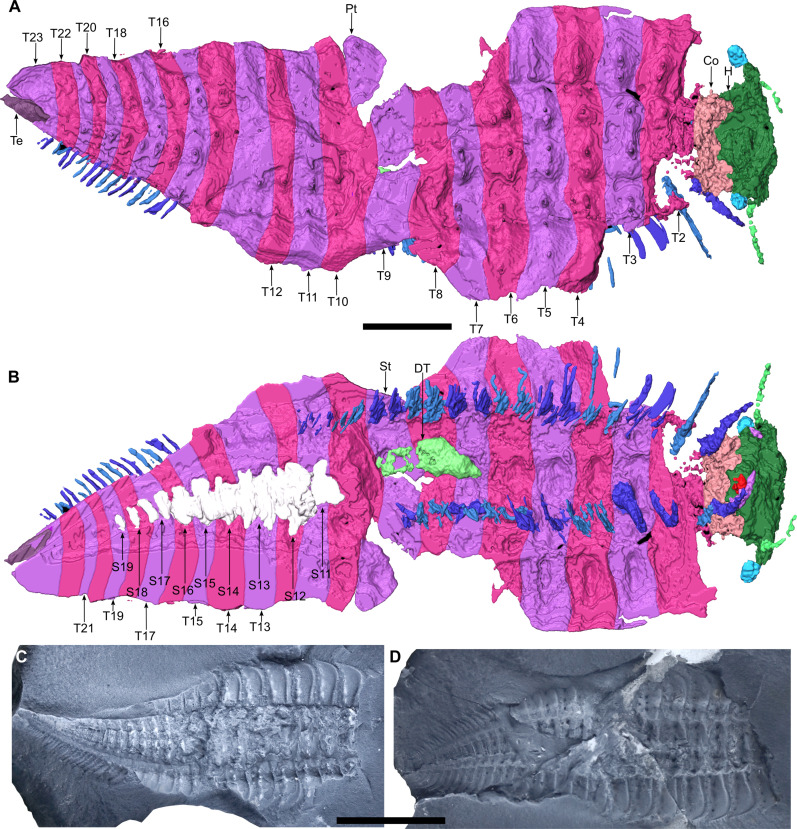
*Arthropleura* sp., specimen MNHN.F.SOT002123. (**A** and **B**) Three-dimensional reconstruction. (A) Dorsal view. (B) Ventral view. (**C** and **D**) specimen inside the nodule. (C) Part. (D) Counterpart. Co, collum; DT, digestive tube; H, head; Pt, paratergite; S#, sternite number; St, syntergite; T#, tergite number; Te, telson. Reconstructions are made from Phoenix X-ray Phoenix V|tome|x CT scan. Scale bars, 1 cm (C and D) and 5 mm (A and B).

**Fig. 2.**
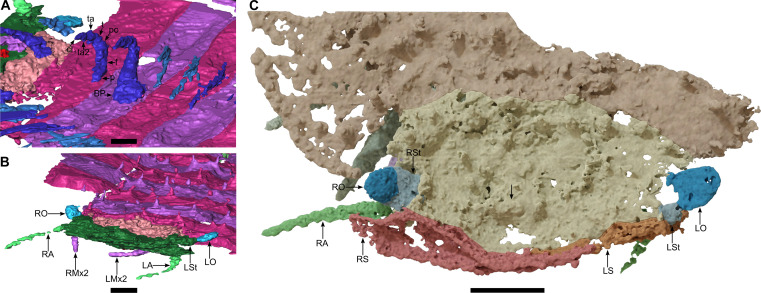
*Arthropleura* sp., specimen MNHN.F.SOT002123, details on the legs and the head. (**A**) Details of the leg. (**B**) Head, three-quarters view. (**C**) Head, dorsal view. BP, B-plate; cl, claw; f, femur; LA, left antenna; LMx2, left second maxilla; LO, left ocular field; LS, left ventral sclerite; LSt, left stalk; p, prefemur; po, postfemur; RA, right antenna; RMx2, right second maxilla; RO, right ocular field; RS, right ventral sclerite; RSt, right stalk; t, tibia; ta, tarsus; ta2, second tarsus. The black arrow on (C) indicates the bulge due to the mandible elements. Panels (A) and (B) are reconstitutions made from Phoenix X-ray Phoenix V|tome|x CT scan. Panel (C) is a reconstruction made from synchrotron x-ray μCT. Scale bars, 2 mm (C) and 1 mm (A and B).

**Fig. 3.**
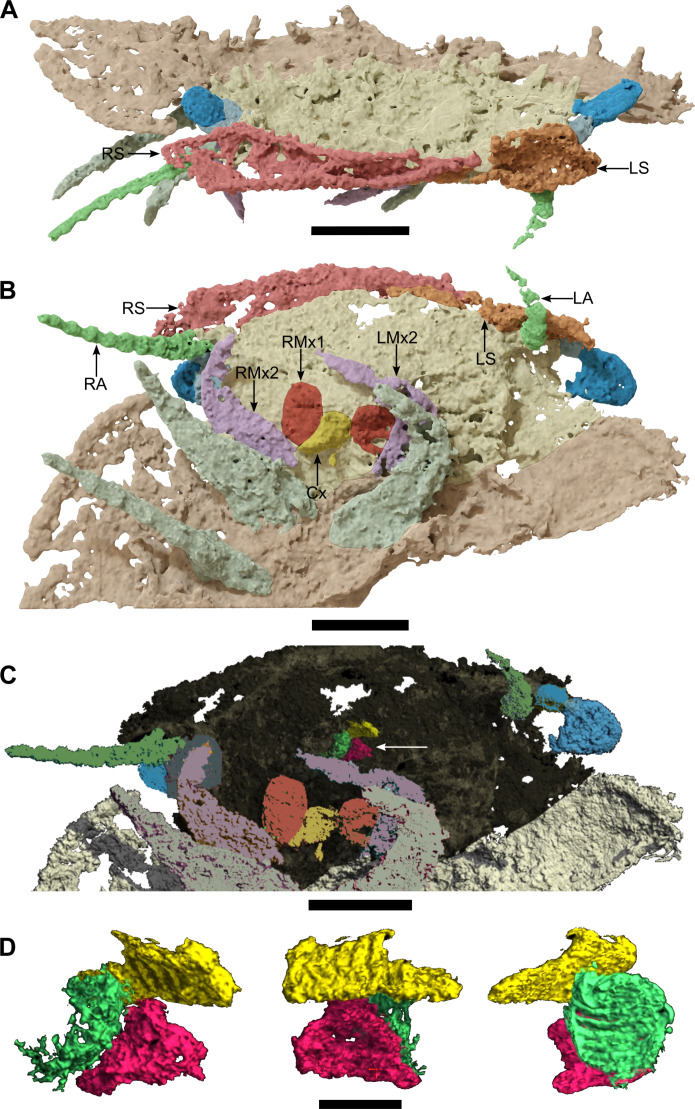
*Arthropleura* sp., specimen MNHN.F.SOT002123, details on the ventral sclerites and the feeding apparatus. (**A**) Head, frontal view. (**B**) Head, ventral view. (**C** and **D**) Mandibles. (C) Localization of the mandible elements. (D) Close-up on the mandible elements, from left to right: ventral, frontal, and left lateral views. Cx, coxosternite; RMx1, right first maxilla. The white arrow on (C) indicates the mandible elements. All reconstructions are made from synchrotron x-ray μCT. Scale bars, 2 mm (A to C) and 400 μm (D).

**Fig. 4.**
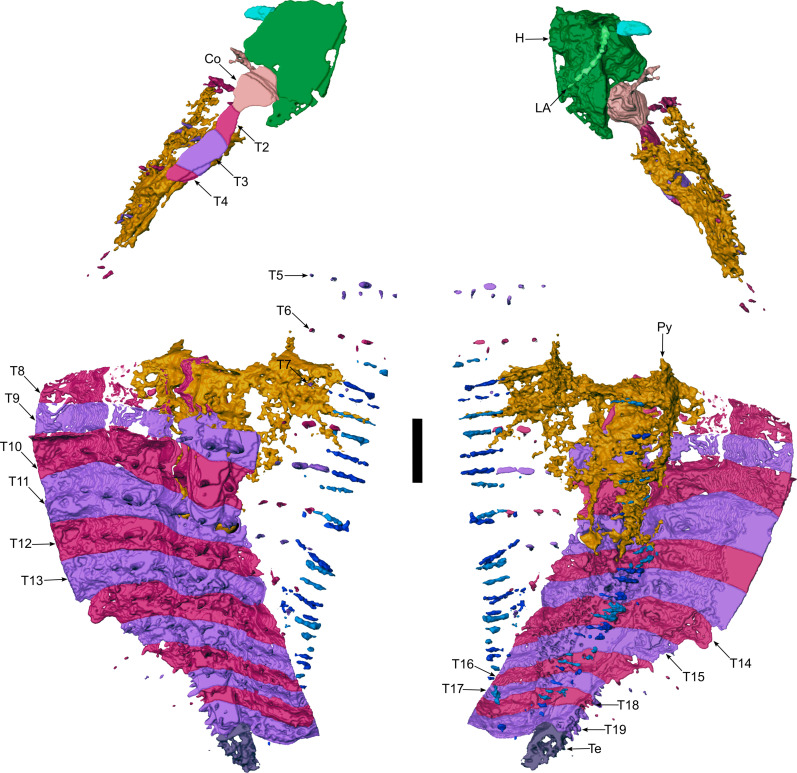
Three-dimensional reconstruction of *Arthropleura* sp., specimen MNHN.F.SOT002118. Py, pyrite. Reconstruction is made from Phoenix X-ray Phoenix V|tome|x CT scan. Scale bar, 2 mm.

**Fig. 5.**
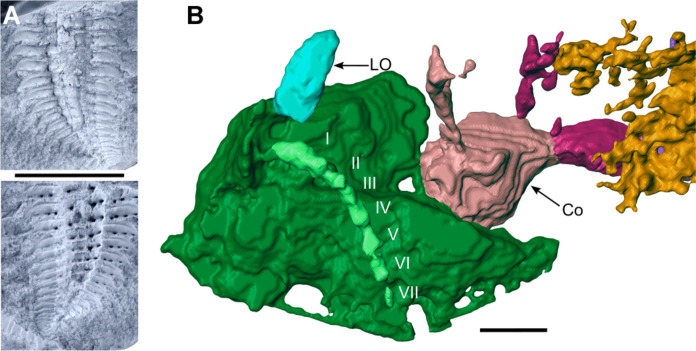
*Arthropleura* sp., specimens MNHN.F.SOT002118, nodule and head. (**A**) Part and counterpart inside the nodule. (**B**) Details on the head. Roman numerals, corresponding antennal articles. Reconstruction in (B) is made from Phoenix X-ray Phoenix V|tome|x CT scan. Scale bars, 1 cm (A) and 1 mm (B).

The complete head is preserved in MNHN.F.SOT002123 (Figs. 1, A and B, 2, and 3). Only the left part is preserved in MNHN.F.SOT002118 (Figs. 4 and 5B). The right ventral cephalic sclerite appears larger than the left due to taphonomic deformation (Figs. 2C and 3A). Stalked eyes are preserved in MNHNF.F.SOT002123 and MNHNF.F.SOT002118 (left only) (Figs. 2C and 5B). A pair of antennae is present in MNHNF.F.SOT002123, inserting ventrally (Fig. 2B and 3, A to C); the left antenna is better preserved in MNHN.F.SOT00218 (Fig. 5B). Elements of the left mandible are present in MNHNF.F.SOT002123. The dorsal exoskeleton is pressed onto distal parts of the mandible (Fig. 3, C and D), presumably taphonomically, creating a bulge on the head in dorsal view (Fig. 2C). Pairs of maxillae are also present in the same specimen (Fig. 3B). The complete trunk is present in MNHN.F.SOT002123. Tergites 5 to 7 for MNHNF.F.SOT002118 were only observed and differentiated because of tubercles. Sternites 11 to 19 are preserved in MNHNF.F.SOT002123 (Fig. 1B). Part of the digestive tract is present below tergites 8 and 9 in MNHNF.F.SOT002123 as a voluminous, non-segmented, and pyritized structure (Fig. 1B). Leg pairs associated with tergites 12 to 14 in MNHN.F.SOT002123 are not preserved. Tergite 8 in MNHN.F.SOT002118 bears only one leg pair, though due to its similarity with the other trunk tergites, we suggest that this is a taphonomic artifact and that the other pair is missing. Leg pairs in MNHN.F.SOT002118 associated with the collum and tergites 2 to 7 are not preserved. Except for the B-plate in MNHN.F.SOT002123, sclerotized plates typical of Arthropleuridea (K- and rosette plates) are not resolved.

### Head

Ventral sclerites are present at the front of the head as large, flattened plates. The eyes are stalked (Figs. 2C and 3A), and ommatida are not observed. The antenna consists of seven articles (Fig. 5B). The first article is larger and oval, while other articles are roughly equal in size and increase in width distally. The mandible with the gnathal lobe is separated from the distal part of the mandible base (Fig. 3D). The coxosternite of the first maxillae is preserved with a telopodite having two podomeres. The coxosternite of the second maxillae is not observed with its telopodite apparently having three podomeres, and a claw is not observed.

### Trunk

**Fig. 6.**
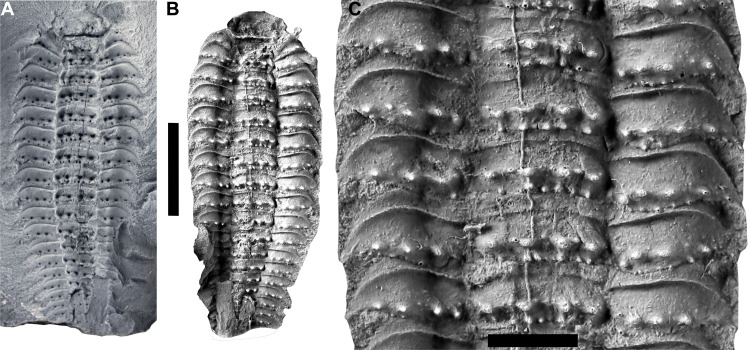
*Arthropleura* sp., specimen MNHN.F.SOT002122. (**A**) Counterpart inside the nodule. (**B**) Latex cast of the counterpart. (**C**) Close-up of ornamentation. Scale bars, 1 cm (A and B) and 3 mm (C).

The trunk consists of a collum, a variable number of somites, and a telson. MNHN.F.SOT002118 bears a total of 20 tergites (Fig. 4), while MNHNF.F.SOT002123 bears a total of 24 tergites (Fig. 1A). The tergite is composed of two parts: a central syntergite bordered on each side by lateral paratergites. The first tergite (collum) is smaller than other tergites and is composed of only a syntergite and no paratergites (Fig. 1A). The terminal body region (telson) has a different shape from the other segments and decreases in width posteriorly (Figs. 1A and 4). Tubercles are present on collum and tergites (Figs. 1A and 4). Syntergites and collum bear two rows of tubercles (Fig. 6). The first row of four tubercles is behind a keel. The second row of four to five smaller tubercles is behind the first one (Figs. 1A, 5A, and 6). Paratergites bear one row of tubercles (homologous to the first row on syntergites). Four tubercles are behind the keel (Figs. 1A, 4, and 6).

### Legs

MNHNF.F.SOT002123 bears 38 pairs of walking legs (Fig. 1A). The collum and the second tergite cover only one pair, while tergites 3 to 11 and 15 to 23 cover two pairs. Tergites 12 to 14 are assumed to cover two pairs of legs each, resulting in a total of 44 pairs of walking legs. MNHN.F.SOT002118 bears 27 pairs of walking legs (Fig. 4). Tergites 9 to 21 cover two pairs. If we suppose that the collum and tergite 2 each had one pair of legs, as in MNHNF.F.SOT002123, and tergites 3 to 8 each had two pairs, then this would result in a total of 40 pairs of walking legs. Each leg consists of eight podomeres, with the terminal podomere being a claw (Fig. 2A). Legs are attached to the sternites by a B-plate (Fig. 2A).

### Phylogenetic results

**Fig. 7.**
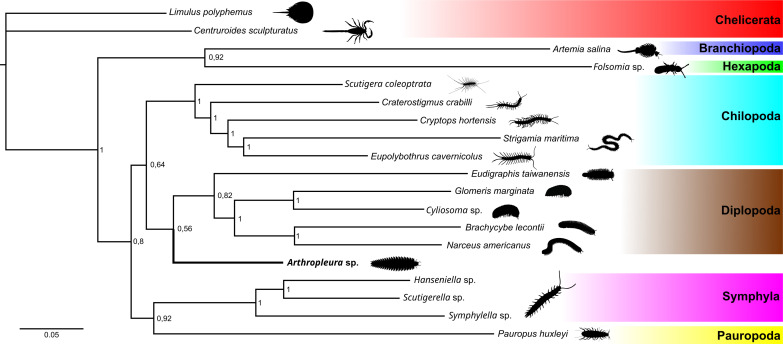
Total-evidence partitioned Bayesian phylogeny. Node numbers are posterior probabilities. The silhouette of *Brachycybe lecontii* was made on the basis of public domain photos. The silhouette of *Arthropleura* sp. was made from the reconstruction in Fig. 8. Other silhouettes from Phylopics.org (attributions in Supplementary Text).

Given the conflicting myriapod trees supported by morphological and molecular datasets (*13*, *14*) and the varied position of *Arthropleura* sp. we obtained through different phylogenetic approaches using just morphological data (figs. S11 to S13), we incorporated a high-occupancy molecular dataset for the extant terminals (designed to minimize missing information) (fig. S13) to infer the placement of *Arthropleura* using a total-evidence Bayesian approach. This analysis recapitulates recent phylotranscriptomic relationships among major myriapod clades, including the monophyly of Edafopoda (Symphyla + Pauropoda) and Pectinopoda (Chilopoda + Diplopoda) (*14*). In this analysis, with *Arthropleura* sp. included as the sole fossil terminal, *Arthropleura* resolves as a stem group millipede (Fig. 7). In a total-evidence Bayesian analysis that also includes the other two arthropleuridean genera *Eoarthropleura* and *Microdecemplex*, *Arthropleura* sp. forms a clade with *Eoarthropleura devonica* that resolves within the myriapod stem group, while *Microdecemplex rolfei* resolves as a chilognathan millipede (fig. S17). To assess the impact that the three fossil terminals have on each other, we analyzed the morphological data after enforcing a molecular backbone constraint for extant arthropods (following fig. S13) and performing all combinations of fossil pruning. These analyses (figs. S14 to S16) show that crown group diplopod affinities for *Microdecemplex* are stable, whereas *Arthropleura* is pulled stemward to the stem group of Pectinopoda by the highly incomplete *Eoarthropleura*, for which more than 71% of characters are coded as missing/inapplicable. We interpret this result as a likely case of biased stemward slippage (*15*).

The clade uniting extant Diplopoda + *Arthropleura* in the total-evidence analysis, shown in Fig. 7, is defined by character 94 (see data S1 for the characters’ different states), which describes diplosegmentation. *Arthropleura* is distinguished from crown group millipedes by having no apical cones on the antennae (character 14), a flattened head capsule (character 21), limbs on the postmaxillary segment (character 66), fused pleurites and tergites to form pleurotergites (character 95), the presence of B- and K-plates (character 187), eight podomeres in the trunk limbs (character 189), and tergites subdivided between a central syntergite and lateral paratergites (character 190). The clade uniting *Eoarthropleura* + *Arthropleura* is defined by fused pleurites and tergites to form pleurotergites (character 95), the presence of B- and K-plates (character 187), and the division of the tergites into a central syntergite and lateral paratergites (character 190). *Arthropleura* sp. may differ from *E. devonica* by having diplosegmentation (character 94); we coded *Eoarthropleura* based on its original description (*4*) but caution that both taxa have alternatively been reconstructed as diplosegmented (*16*). The crown group diplopod clade uniting Helminthomorpha + *M. rolfei* shares fused pleurites and tergites to form pleurotergites (character 95).

## DISCUSSION

### Phylogenetic affinities

**Fig. 8.**
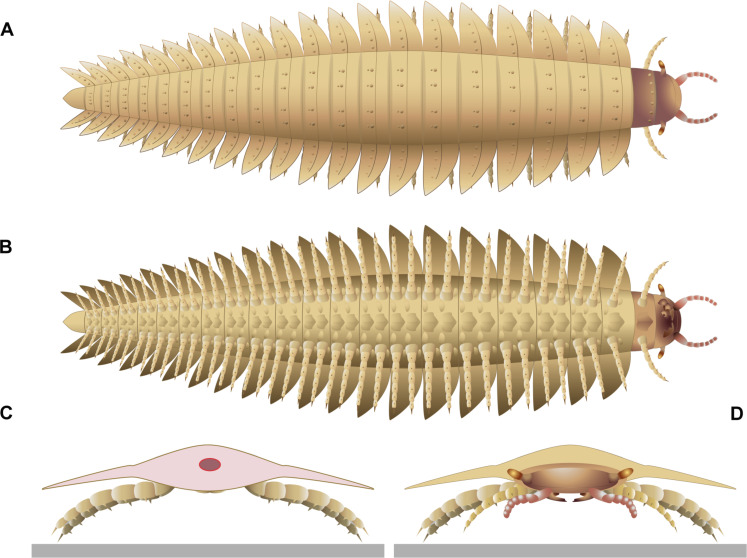
Reconstruction of specimen MNHNF.F.SOT002123. (**A**) Dorsal view. (**B**) Ventral view. (**C**) Back view. (**D**) Frontal view. Left maxillae were removed on (B) to better illustrate the mandible below. The red circle on (C) indicates the position of the digestive tract.

The subphylum Myriapoda consists of four classes: Chilopoda (centipedes), Symphyla (garden centipedes), Pauropoda, and Diplopoda (millipedes). Strict diplosegmentation, with two pairs of legs per tergite, is characteristic of Diplopoda. Although the *Arthropleura* specimens present synapomorphies characteristic of extant millipedes such as a clear diplosegmentation and a modified tergite between the head and the trunk (i.e., a collum) (Fig. 1, A and B), the fossils also have a head morphology with some characters akin to those found among centipedes. Most notably, the feeding apparatus includes first maxillae that are small, with blunt lobes corresponding to a coxal process and short telopodite (Figs. 3B and 8) and do not correspond to a plate-like structure similar to the diplopod gnathochilarium (fig. S9), a specialization of the millipede first maxilla. The second maxillae are leg-like and seem to have three podomeres in their telopodite (details are uncertain because of the difficulty of recognizing the podomere boundaries; Fig. 3B). This is shared by many centipedes and contrasts notably with the absence of an appendage on the second maxillary segment in Diplopoda (fig. S9) and Pauropoda. The mandibles, on the other hand, resemble those of Diplopoda in having a separated gnathal lobe and mandibular base (of which only the distal part is observed here; Fig. 3D). This contrasts with the mandibles of Chilopoda which have a weakly delineated separation between their thin and curved base and their flattened and large gnathal lobe (fig. S9) (*17*, *18*). However, the mandible is fully encapsulated within the head (as in centipedes) (Fig. 8) rather than having its base plate forming the lateral wall of the head capsule as is the case in diplopods and symphylans. Other appendages of *Arthropleura* sp. are millipede-like, notably antennae with seven articles (Figs. 3B and 5B).

The combination of millipede trunk tagmosis with centipede-like head characters such as leg-like second maxillae is challenging to reconcile with traditional myriapod morphological phylogenies, which have always supported a distant relationship between Chilopoda and Diplopoda (*13*). However, recent molecular phylogenies (*14*) have changed this view, instead placing Chilopoda and Diplopoda as sister groups within the clade Pectinopoda. This grouping (and the evolutionary scenario linked to it) is consistent with *Arthropleura*’s anatomy, illustrated by our phylogenetic analyses that place *Arthropleura* inside Pectinopoda, more closely allied to millipedes than to centipedes (Fig. 7 and figs. S15B and S16C). While other alternatives are recovered, including placements as a stem group pectinopodan (fig. S15C) and as a stem group myriapod (fig. S17), these may be biased by missing data. Regardless of this, all possible placements of *Arthropleura* imply that the morphological features it shares with centipedes are plesiomorphic conditions of pectinopodans. Although *E. devonica* has rampant missing data due to the fragmentary preservation of its specimens, it still displays apomorphic characters shared with *Arthropleura* like the presence of B-plate and K-plate and the division of its tergites into syntergites and paratergites, and it can be grouped with *Arthropleura* in the subclass Arthropleuridea (figs. S11B, S14, S15C, and S17). On the contrary, *Microdecemplex rolfei* has characters typical of crown group millipedes such as a diplopody and pleurotergites, and it is consistently separate from the arthropleurideans across our phylogenetic analyses (figs. S11B, S14, S15, A and B, S16A, and S17). We accordingly identify the order Microdecemplicida as Diplopoda/Chilognatha rather than Arthropleuridea.

Our findings on *Arthropleura* also illuminate the evolutionary steps involved in the acquisition of the unique morphologies that characterize the body plan of extant millipedes, such as the anatomy of the collum. Extant millipedes lack legs on the collum, while our scans suggest that *Arthropleura* sp. bears one pair (Figs. 1A and 8). This has the following implications: (i) that the loss of legs on the collum did not appear at the same time as diplosegmentation (present in *Arthropleura*) but is rather a condition restricted to crown group millipedes and (ii) the presence of legs on the collum in arthropleurids also implies that the collum originated as a trunk segment (*19*).

In contrast with extant myriapods that have sessile lateral eyes (*20*), *Arthropleura* sp. has stalked eyes (Figs. 2, B and C, 3A, 5B, and 8), a morphology that is more compatible with the eyes being compound, as in scutigeromorph centipedes and penicillate millipedes (*21*), rather than being a cluster of stemmata that are sessile on the head, as in the remainder of Chilopoda and Diplopoda (fig. S9). The only total group myriapods with stalked compound eyes are the extinct, possibly amphibious Euthycarcinoidea (Cambrian-Triassic), now considered to be part of the myriapod stem group (*22*).

### Feeding and locomotion

Diet in Myriapoda is separated between the carnivorous Chilopoda and the detritivorous Symphyla, Pauropoda, and Diplopoda. The diet of *Arthropleura* remains an open question as few appendages of the head and essentially none of the feeding apparatus have previously been found. Although no direct evidence of its diet has been found, a detritivorous diet is supported by most authors [see, e.g., (*5*, *6*, *18*)] based on elements preserved inside the gut of one specimen (*23*) (later reidentified as a taphonomic artifact) (*8*) and on suppositions based on their putative phylogenetic placement within millipedes (which are nearly all detritivorous) (*8*). Even in the light of our results that some aspects of the group’s feeding apparatus are more similar to those of carnivorous centipedes (fig. S9), the overall anatomy of *Arthropleura* suggests that it was likely a detritivorous myriapod. For instance, *Arthropleura* sp. lacks forcipules (a first pair of trunk appendages modified for venom delivery) which are present in the predatory centipedes, and its post-mandibular cephalic limbs are not modified to catch prey, as is seen in the predatory arachnids. The diplosegmentation and short, millipede-like locomotory limbs imply at most moderate rates of locomotion through comparison with living taxa (*24*) and preclude the fast backstroke seen in centipedes. No hints of two series and ornamented (shape of the appendage tips visible) stroke typical of fast-moving centipedes (*25*) are observed on trackways present in Montceau (fig. S8) or other *Diplichnites cuithensis* from different localities (*26*–*29*), further suggesting slow locomotion. Hence, we consider it likely, pending further clear hints of its diet such as gut remains, that *Arthropleura* was a slow detritivorous myriapod.

### Ontogeny

Postembryonic development in myriapods is usually classified into two types: epimorphic, when the individual has a fixed number of segments and increases only in size with each molt; and anamorphic, when the individual adds segments and increases in size with each molt. The two types are observed in centipedes but are invariant at the ordinal level (*30*). All millipedes have anamorphic development with different subcategories: (i) euanamorphic, with the addition of segments at each molt even after sexual maturity; (ii) hemianamorphic, where individuals reach a fixed maximum number of segments, after which, a few epimorphic molts follow but stop with sexual maturity; and (iii) teloanamorphic, which reaches a maximum segment number, followed by sexual maturity and no epimorphic molts afterward (*31*).

The *Arthropleura* specimens of Montceau show juvenile features that are consistent with hemianamorphic development. Specimen MNHNF.F.SOT002123, which bears more segments (24 segments) than MNHNF.F.SOT002118 (20 segments), is also larger (body lengths of 39.8 mm for MNHN.F.SOT002123 and 23.1 mm for MNHN.F.SOT002118) (table S1) suggesting that, under a hemianamorphic hypothesis, MNHNF.F.SOT002123 is older (and thus molted more times) than MNHNF.F.SOT002118. In both specimens, the posterior tergites and leg pairs are smaller than their anterior counterparts (Figs. 1A and 4) presumably because they are still in development. This implies that *Arthropleura* gained segments and leg pairs with each molt. Trackways of bigger specimens in Montceau-les-Mines (5 cm wide, associated with specimens probably reaching 40 cm in length) (fig. S8) (*32*) show that the specimens of *Arthropleura* sp. preserved in the nodules are not at their maximal length and width. This implies a taphonomic bias in the size of the arthropleurids preserved in this way—with further molts, we could expect these individuals to increase in size. The Montceau trackways were probably formed by larger representatives of the same taxon as the juveniles.

The pattern of ornamentation in Montceau specimens is very similar to that of *Arthropleura moyseyi* (found in the United Kingdom during the Bashkirian) (*33*) and larger *Arthropleura mammata* specimens found in the United Kingdom, France, and Belgium during the Bashkirian (figs. S1, S2, S6, and S7) (*34*–*37*). Both the Montceau specimens and *A. mammata* exhibit a row of four large tubercles just posterior to the keel on the paratergites (figs. S6 and S7). Montceau specimens and *A. moyseyi* both exhibit the same pattern on the paratergites and the syntergites, with a second row of three smaller tubercles behind the first row of large tubercles (figs. S6 and S7). Because of the large temporal gap between the Montceau specimens and *A. mammata* and *A. moyseyi* (roughly 10 million years), we can infer that even if they have the same ornamentation patterns, the Montceau specimens are different species. This is also another argument against the use of ornamentation patterns as a distinction for *Arthropleura* species; they may instead merely be “morphotypes” (see Supplementary Text for more details on morphotypes). It seems likely that juvenile *Arthropleura* would follow a hemianamorphic development. It would be during the epimorphic stages that the animal reached its famous gigantic size as seen in species such as *Arthropleura armata* (fig. S5). This hypothesis informs on the plesiomorphic developmental condition in millipedes and centipedes. A placement of *Arthropleura* within Pectinopoda [which groups centipedes, in which anamorphic development is plesiomorphic, and millipedes, which are all anamorphic (*30*)] implies that the ancestral state for pectinopodans is anamorphic development. Furthermore, this pertains to myriapods as a whole, as all Edafopoda (i.e., symphylans and pauropods) likewise have anamorphic development (*13*).

Concerning the size of adult *Arthropleura* sp. from Montceau, it is possible that the Montceau adults may not have been gigantic and could instead be a previously unknown species and a small representative of the genus. The absence of remains of large individuals (either large tergites or trackways) and the biggest trackway ever found being associated with an individual smaller than 1 m long may suggest that gigantism is not a universal condition present in the genus *Arthropleura* and that this group could have had both giant and small representatives.

### Unresolved questions

Even after gaining insight into their head morphology, there remain unanswered questions about arthropleurids, especially their ecology. The Montceau specimens’ digestive tracts do not reveal any food remains that can help identify their diet. No remains of a respiratory system were found, so the presence of either tracheae as in extant myriapods or branchial lamellae as in extant aquatic arthropods, and thus, the relation between *Arthropleura* and water cannot be ascertained. The stalked eyes found in Montceau specimens could point toward a semi-aquatic lifestyle, as this peculiarity is shared with the amphibious euthycarcinoids which are also present in the Montceau-les-Mines Lagerstätte (*38*). Questions remain regarding these stalked eyes which seem to be an evolutionary reversal found within the myriapod crown group only in Arthropleurida. It is also worth noting that there is no direct evidence that the adult conspecifics of Montceau juveniles were giant (e.g., no remains of giant specimens were found although this could be a taphonomic bias, as the nodules of Montceau are never big enough to preserve such large specimens, even partially). The uncertainty on the ontogenetic stages of *Arthropleura* underscores our lack of knowledge of other aspects of their biology, such as their growth rate and possible sexual dimorphism.

Noninvasive μCT analysis of *Arthropleura* sp. has revealed the first insight into limbs of the head in this genus of gigantic myriapods, as well as details on trunk anatomy, such as the terminal segments showing the presence of a telson, based on a complete specimen. Multiple clues suggest that the Montceau-les-Mines arthropleurids represent juveniles. Their morphology (decrease in size of the last tergites and leg pairs posteriorly) and their relationship with extant millipedes and centipedes corroborate previous theories of hemianamorphic growth being the plesiomorphic type of development in myriapods. *Arthropleura* sp. displays a mix of characters from different myriapod classes, including a trunk having derived characters similar to extant Diplopoda (the presence of a collum and diplosegmentation, antennae with seven segments, and mandibles composed of a separated base plate and gnathal lobe) and a head with some characters found in Chilopoda (fully encapsulated mandibles and small pair of first maxillae followed by a pair of leg-like second maxillae). This morphology is consistent with the evolutionary scenarios implied by recent molecular phylogenies (*14*), which group Chilopoda and Diplopoda within Pectinopoda, and our total-evidence phylogenetics accommodates this hypothesis.

## MATERIALS AND METHODS

### Fossil material

We studied the seven specimens identified as *Arthropleura* in the Autun Museum of Natural History: MNHN.F.SOT002118, MNHN.F.SOT002119, MNHN.F.SOT002122, MNHN.F.SOT002123, MNHN.F.SOT002124, MNHN.F.SOT003983, and MNHN.F.SOT076155. All of them were collected from the Saint-Louis site except for MNHN.F.SOT076155 whose exact provenance is not specified although it is clearly from the Montceau-les-Mines Lagerstätte. MNHN.F.SOT002124 is not an arthropleurid and belongs to *Amynilyspes fatimae* (Diplopoda) (*39*). The two best-preserved specimens are presented in this study: MNHN.F.SOT002118 (Figs. 4 and 5) and MNHN.F.SOT002123 (Figs. 1 to 3). Specimen MNHN.F.SOT002122 is also figured as it is the specimen with the best-preserved ornamentation (Fig. 6). The remaining material is poorly preserved and provides no key information on the appendages or soft anatomy. All studied specimens are property of the Museum National d’Histoire Naturelle of Paris (MNHN) and are deposited in the collections of the Museum d’Histoire Naturelle d’Autun under the Sotty collection (number MNHN.F.SOT).

### Imaging methods

All specimens were photographed with a Canon EOS 5D SR camera equipped with a Canon 100-mm macro lens after being whitened with ammonium chloride. A latex cast of MNHN.F.SOT002122 was realized to better visualize ornamentation. μCT data were obtained with a GE Sensing & Inspection Technologies Phoenix V|tome|x CT-scanner at the Matériaux Ingénierie et Science (MatéIS) Laboratory of the Institut National des Sciences Appliquées Lyon (INSA Lyon). The source was operated at 140 kV with a current of 80 μA and a copper filter of 0.5 mm thickness for all specimens. Each scan consisted of 1000 projections with an exposure time of 1 s and averaged three radiographs for each projection. Voxel size was 40 μm for MNHN.F.SOT002123 and 30 μm for MNHN.F.SOT002118. The cone beam μCT data were reconstructed by a standard filtered back projection Feldkamp algorithm. Three-dimensional (3D) models are stored in the MorphoMuseuM journal (*40*). Specimen MNHNF.F.SOT002123 was also imaged using propagation phase contrast synchrotron x-ray μCT on the BM18 beamline of the European Synchrotron Radiation Facility (ESRF; Grenoble, France). The beamline configuration consisted of white beam from a triple wiggler (central pole at 1.56 T, two lateral poles at 0.85 T, fixed gap) filtered with W 0.3 mm and Mo 3.75 mm, resulting in an averaged detected energy of ~180 keV; indirect detector comprising a 250-μm LuAG:Ce crystal scintillator, a visible light zoom lens (variable magnification from ×1 to ×5) and an Iris 15 sCMOS camera (Teledyne Photometrics, Tucson, AZ, USA) generating data with a voxel size of 3.04 μm. The sample detector distance was 4 m for phase contrast images. The acquisition consisted of 14,000 projections, each with a total exposure time of 210 ms (accumulation of three images of 70 ms), continuous rotation over 360°, 41 flatfield images (image of the beam with no sample), and 40 dark current images (images with no x-ray beam). Given the size of the sample and the limited field of view at this resolution (*v* × *h*: 2304 × 5056 pixels, ~7 × 15.5 mm), 12 acquisitions were necessary, moving the specimen on the vertical axis of the sample stage between each scan by 3.5 mm. Moving by 50% of the vertical field of view allowed limited artifacts induced by the vertical intensity profile of the beam. In addition, an offset of 6.08 mm (corresponding to 2000 pixels on the detector) was applied to the center of rotation, allowing an increase of resolution in the reconstructed slices by 4000 pixels through projections recorded 180° apart. Tomographic reconstruction was performed using PyHST2 and the single distance phase retrieval approach (*41*, *42*). The delta/beta value was set to 500 as a priori knowledge for the phase retrieval. Postprocessing of the 32-bit data included the following: stitching of the dataset along the vertical axis using weighted averaging; ring correction (*43*); change of the dynamic range to 16 bits based on 0.001% saturation values of the 32-bit histograms; cropping of the data as close as possible to the specimen preserved in the nodule; binning of the data (2 × 2 × 2 in bicubic approach); and export of the final complete stack at 16-bit tiff images. MATLAB codes used for ring correction, binning, and cropping can be found at https://github.com/HiPCTProject/Tomo_Recon. Most tomograms were segmented with Avizo and SPIERS v. 3.1.0 software (*44*). The 3-μm dataset of the head of MNHN.F.SOT002123 was segmented with VGSTUDIO MAX 2023 (Volume Graphics, Heidelberg, Germany) using the “paint and segment” tools (i.e., deep learning approach). About eight iterations of training and adjusting input painting data were necessary to achieve a result considered visually satisfactory. Minor adjustments were made using various tools of the software to refine the segmentation (e.g., erode/dilate, smoothing, refining, and region growing while staying in the region of interest). Anatomical parts were segmented manually using information from the tomogram and the live 3D rendering. Each part was extracted individually and converted into a 3D mesh model using the grid-based watertight approach with no simplification. Simplification and rendering of the 3D mesh models were done using Blender 3.6 (Blender, Amsterdam, Netherlands). The simplification comprised a series of modifiers (decimate with a factor of 0.5, smoothing with a factor of 2, remeshing voxel with a size of 6 μm). DOI for the raw tomographic data is 10.15151/ESRF-DC-1750892610.

### Phylogenetic analyses

The relationships between *Arthropleura* sp. and other extant myriapods were studied using two approaches. First, a morphological analysis using a pre-existing matrix was conducted to place *Arthropleura* sp. in the framework of “classical” myriapod phylogeny (i.e., Progoneata and Dignatha) (*45*). Then, molecular data from the phylotranscriptomic analysis of Benavides *et al*. (*14*) were added to further constrain the placement of *Arthropleura* and investigate character evolution in the phylogenetic context proposed by Benavides *et al*. (Edafopoda and Pectinopoda). Then, the same approach was made, adding the two other taxa previously considered members of Arthropleuridea: Eoarthropleurida with *E. devonica* and Microdecemplicida with *M. rolfei*. All datasets were analyzed using maximum parsimony (MP), maximum likelihood (ML), and Bayesian inference (BI).

#### Morphological data and inferences

Nineteen species of extant arthropods were analyzed, including 15 myriapod species (14 extant species + *Arthropleura* sp.). Characters and taxa are mostly from Fernández *et al*. (*13*). Two chelicerate species, *Limulus polyphemus* and *Centruroides sculpturatus*, were coded and included as outgroups. The final data matrix is composed of 190 characters, including 8 ontogenetic characters, 68 head characters, 74 body characters, 31 sexual characters, and 9 other characters, 3 of which were not present in the original data matrix and were added by the authors. Character descriptions are included in data S1. The MP analysis (unordered multistate, with an exhaustive search algorithm) was made using TNT v.1.5 (*46*). Consistency index, homoplasy index, retention index, and rescaled consistency index were calculated, as well as nonparametric bootstrap supports (1000 replicates). Results were summarized using a strict consensus tree. Inference was repeated after removing *Arthropleura* to evaluate its effect on levels of resolution. Character mapping was performed in TNT to identify unambiguous synapomorphies.

BI of the morphological dataset was performed using MrBayes v3.2.7a (*47*) under an M*k*_v_ + Г model (*48*). Four runs of four chains each were continued for 20 million generations, sampling every 500 and discarding the initial 25% as burn-in. Convergence and stationarity were visually confirmed using Tracer v1.7.1 (*49*); the average SD of split frequencies was 0.001, and all parameters attained potential scale reduction factors of 1.0.

#### Molecular data and inferences

We took the high-occupancy version (matrix M4: 101 loci, 30,728 amino acids) of the latest phylogenomic dataset of myriapods (*14*) and pruned the terminals down to the 18 coded for morphology. Positions with more than 30% missing entries were trimmed, and the remaining loci were subsampled to those with more than 80% occupancy. The final dataset was composed of 51 loci and 14,570 positions. A best-fit partitioned model was determined using IQ-TREE v2.0.3 (*50*–*52*), allowing loci to be merged and constraining model choice to those available in MrBayes. An ML tree using the optimal partitioned model was also obtained using IQ-TREE2, estimating support values with 1000 replicates of ultrafast bootstrap (*53*). The resulting topology (fig. S13) was identical to that of Benavides *et al*. (*14*) apart from one node in Chilopoda (Lithobiomorpha + Geophilomorpha).

#### Total-evidence data and inferences

The molecular dataset (subdivided into 12 partitions) was combined with the morphological dataset (analyzed as a single partition) for total-evidence BI under the same conditions as mentioned above. Convergence was evaluated (and confirmed) using the same approach.

#### Constrained total-evidence analyses

We also ran taxon depletion experiments using a constrained tree topology. For this, we ran the morphological dataset while enforcing a molecular backbone topology reflecting the inferred relationships of extant taxa in the phylogenomic dataset (as obtained under ML; fig. S13). This used soft constraints to fix the relationships among extant terminals while leaving the position of fossils free to be estimated. We did this for all seven possible combinations of the three arthropleuridean fossils of interest.

## Supplementary Materials

### The PDF file includes:

Supplementary Text Figs. S1 to S17 Tables S1 to S9 Legends for data S1 to S7 References
