# Supplementary material for: Head anatomy and phylogenomics show the Carboniferous giant Arthropleura belonged to a millipede-centipede group
Source: Sci Adv. 2024 Oct 9;10(41):eadp6362. doi: 10.1126/sciadv.adp6362 (PMC11463278; doi:10.1126/sciadv.adp6362)
Supplement: Supplementary file 1 — Supplementary Text Figs. S1 to S17 Tables S1 to S9 Legends for data S1 to S7 References [file sciadv.adp6362_sm.pdf]

Science Advances
AAAS

Supplementary Materials for

Head anatomy and phylogenomics show the Carboniferous giant *Arthropleura* belonged to a millipede-centipede group

Mickaël Lhéritier *et al.*

Corresponding author: Mickaël Lhéritier, mickael.lheritier@univ-lyon1.fr

Sci. Adv. **10**, eadp6362 (2024)
DOI: 10.1126/sciadv.adp6362

The PDF file includes:

Supplementary Text
Figs. S1 to S17
Tables S1 to S9
Legends for data S1 to S7
References

Other Supplementary Material for this manuscript includes the following:

Data S1 to S7

Supplementary Text

Systematics of the Arthropleurids of Montceau-les-Mines

Subphylum **Myriapoda** Latreille, 1802
Total-group **Diplopoda** de Blainville in Gervais, 1844 or total-group **Pectinopoda** Benavides, Edgecombe and Giribet, 2023
Subclass **Arthropleuridea** Waterlot, 1934

Diagnosis (emended from Edgecombe in Minelli, 2015). Myriapods with paratergal lobes separated from the syntergal axis by sutures. Sclerotized plates (K, B and rosette plates) buttressing the leg.

Comparison. *Eoarthropleura* and *Arthropleura* share non calcified cuticles. Both display ventral sclerites known as a B-plate and a K-plate, and a clear separation of tergites between a central syntergite and lateral paratergites. As seen in MNHN.F.SOT002123 and MNHN.F.SOT002118, *Arthropleura* bears diplosegments from tergite 2 to the penultimate tergite while *Eoarthropleura* was originally described with one pair of legs for each tergite (*4*), but has also been considered diplosegmented (*16*).

Microdecemplex and *Arthropleura* share a non-calcified cuticle, and diplosegmentation. *Arthropleura* displays a flattened head capsule while that of *Microdecemplex* is domed. The collum of *Microdecemplex* is apodous while our tomography suggests that *Arthropleura* had a pair of legs. *Microdecemplex* does not have K-plates and its tergites are not separated between syntergites and paratergites. The legs in the studied *Arthropleura* specimens possess eight podomeres whilst those of *Microdecemplex* bear seven. However, a range of 7-11 podomeres has been reported from different *Arthropleura* specimens (*16*). Contrary to *Arthropleura*, *Microdecemplex* as a sternal intermaxillary plate on the first maxillary segment like that of the diplopod gnathochilarium.

Discussion. The presence of an apodous collum has previously been attributed to Arthropleuridea and considered a synapomorphy of a clade that unites Diplopoda and

Pauropoda (Dignatha) (*5*) but the presence of a leg pair beneath the collum tergite in *Arthropleura* sp. specimen MNHN.F.SOT002123 (Figs 1B and 4) contradicts this character. Characters based on the paratergal lobes (separated from the axis by sutures) and the sclerotized ventral plates at the legs bases concern only Eoarthropleurida and Arthropleurida and are not valid for Microdecemplicida, which is known from a single Middle Devonian species that has variably been assigned to Arthropleuridea (*11*). Monophyly of Arthropleuridea including *Microdecemplex* is supported only by a lateral embayment in the collum, and the alternative that this grouping is paraphyletic was suggested by Kraus & Brauckmann (*5*). We propose to assign only Eoarthropleurida and Arthropleurida to the subclass Arthropleuridea to make it a putatively monophyletic group with synapomorphies being a differentiation of the tergites in syntergites and paratergites and the presence of different sclerotized ventral plates (K, B and rosette plates). Microdecemplicida has previously been placed in the diplopod crown group as most closely related to Chilognatha (*3, 16*), while our phylogenetic analysis places it in the Chilognatha crown-group (figs. S12B, S15, S16A, B, S17A, S18).

Order **Arthropleurida** Waterlot, 1934

Diagnosis (emended from Edgecombe in Minelli, 2015) (*11, 12*). Arthropleuridea with at least 23 trilobed dorsal plates bearing irregular large and small tubercles.

Remarks. Although Kraus & Brauckmann (*5*) counted 29 trilobed paratergal plates in the Montceau specimens, the present restudy of the same material using µCT indicates that *Arthropleura* sp. has no more than 23 paratergal plates. However this count is made on in what we consider juvenile specimens (for discussion on juvenile/small species, see section Ontogeny). Given anamorphic growth (post-embryonic addition of segments, see section Ontogeny) in arthropleurids, this is not the ultimate number of paratergal plates in adults.

Family **Arthropleuridae** Von Zittel, 1885

Diagnosis. Same as order.

Remarks. Monogeneric family.

Genus ***Arthropleura*** Jordan in Jordan and Von Meyer, 1854

Type species. *Arthropleura armata*. Holotype K $_{13\text{-}14}$ in the Museum für Naturkunde, Berlin (fig. S5).

Diagnosis. Same as family.

***Arthropleura* sp.** Jordan in Jordan and Von Meyer, 1854
(figures 1-5)
1980 Arthropleurida Secretan (*54*): 27, pl. II, figs. 3-7.
1985 Arthropleurida Almond (*10*)
1994 Briggs & Almond (*9*): 128-130, figs. 1-2.
2003 *Arthropleura* Kraus & Brauckmann (*5*) : 13, fig. 4.

Discussion. Species within *Arthropleura* are differentiated by their tergal ornamentation patterns. Due to the scarcity of significantly complete specimens and the palaeogeographical proximity of all species (found in western Germany, western Belgium, northern and central France and various part of England) except for *Arthropleura cristata* Richardson 1959 (found in the United Sates), we suggest that some nominal species could be just different morphotypes and could be regrouped within a single species. It is difficult to identify clear interspecific variations, and differences in ornamentation pattern could be due to intraspecific variation, ontogenetic variation or sexual dimorphism. The type species, *Arthropleura armata* Jordan and Von Meyer 1854, is distinguished mainly by the arrangement of the paratergal tubercles. *Arthropleura mailleuxi* Pruvost 1930 (*34*) found in Belgium and France (see fig. S1 for the occurrences of the different *Arthropleura* sp.), is also very tuberculated but less so than *A. armata* with most of the ornament on the antero-lateral part of the paratergite behind the keel. *Arthropleura fayoli* Boule 1893 (*55*) is found in France. Its pattern consists of a very granulated furrow, with no tubercles behind the keel and a posterior part with two large tubercles and two smaller tubercles (from the most distal to the most proximal part of the

paratergite). This is followed by another, posterior series of three or four small tubercles. Our observations on Montceau specimens show similar patterns with *Arthropleura mammata* Salter 1863 (*35*) and *A. moyseyi* Calman 1914 (*33*) (more details in Ontogeny section). *Arthropleura britannica* Andrée 1913 (*56*), from Belgium is very similar to *A. mammata* (two bands of tubercles behind the keel) and *A. fayoli* (size variation of the tubercles within the same range). *Arthropleura cristata* from the Mazon Creek biota (USA) is peculiar in lacking any ornament. This character and its distant paleogeographic position (it is the only *Arthropleura* species described in North America) lead us to think that *A. cristata* belongs to a different species than the Montceau specimens. The only morphotypes contemporary with Montceau specimens are *A. armata* and *A. britannica* (fig. S3). The Montceau specimens differ remarkedly from these two nominal taxa in terms of ornamentation pattern and size, suggestive of the individuals of Montceau being a possible new species. However, because of the scarcity of good characters to define clear *Arthropleura* species, many workers do not make specific assignments and describe their specimens in open nomenclature as *Arthropleura* sp. For the same reason, we notice the similarity and differences of the Montceau specimens with some morphotypes but we do not synonymize them or establish a new species. On a side note, the similar morphology (notably on the posterior tergites) of *A. moyseyi* and Montceau specimens helps us to characterize the specimen of *A. moyseyi* as a juvenile, possibly of *A. mammata* as the two morphotypes are similar and were found in close locations in term of palaeogeography.

Details on morphology only analyses

Phylogenetic inference of morphological data under maximum parsimony (MP) returns five most parsimonious trees (fig. S10). In their strict consensus (fig. S10F), *Arthropleura* sp. forms part of a polytomy that also includes the sampled pancrustacean taxa, as well as five

lineages of crown-group myriapods: the only sampled pauropod *Pauropus huxleyi*, the penicillate diplopod *Eudigraphis taiwanensis*, Symphyla, the remainder of Diplopoda (Chilognatha), and Chilopoda. A very similar result is found under Bayesian inference (BI; fig. S11): although with less polytomy, the position of *Arthropleura* relative to extant myriapods and pancrustaceans is unresolved. This instability is driven in part by *Arthropleura* itself but also by homoplasy between extant taxa in the matrix. Removal of *Arthropleura* from the matrix and reanalysis under MP results in two most parsimonious trees (fig. S12). In the first tree, we obtain a topology with myriapod monophyly (fig. S12A) while in the second tree the collembolan *Folsomia* sp. is grouped with Pauropoda and Diplopoda (fig. S12B) because of two antennal characters: a fixed number of antennal articles within the species (character 10), and eight or fewer articles (character 11). Its combination of features typical of different crown-group myriapod clades may contribute to *Arthropleura* behaving as a rogue terminal. However, we note that a close affinity between *Arthropleura* and millipedes (figure *S*11) is recovered in four of five most parsimonious trees, consistent with the result we obtain in total evidence analysis (Fig. 7).

Supplementary information on character 95 (presence of pleurotergites)

Character 95 (presence of pleurotergites) is difficult to confirm as no distinct pleurites were observed due to taphonomic/preparation biases. Still, there are examples of Palaeozoic millipedes where distinct pleurites were not observed yet numerous morphological characters indicate a close similarity with extant Pentazonia millipedes, suggesting they should have this character state (*39*), a condition that likely applies to our three fossil taxa as well. The only explicit evidence for pleurites in *Arthropleura* is discussed in Kraus & Brauckmann (2003, pp 14-17) (*5*).

Phylopics silhouettes attributions

Chelicerata: -*Limulus polyphemus*: Andy Wilson
-*Centruroides*: Christoph Schomburg

Branchiopoda: -*Artemia salina*: Thomas Hegna

Hexapoda: -*Folsomia candida*: Kamil S. Jaron

Pauropoda: -*Pauropus huxleyi*: Gemma Martínez-Redondo

Symphyla: -*Scutigerella immaculata*: Gemma Martínez-Redondo

Chilopoda: -*Scutigera coleoptrata*: Guillaume Dera
-Craterostigmomorpha (order comprising *Craterostigmus crabilli*): Gemma Martínez-Redondo
-*Scolopendra gigantea* (order Scolopendromorpha, same order and similar morphology as *Cryptops hortensis*): Guillaume Dera
-Geophilomorpha (order comprising *Strigamia maritima*): Gemma Martínez-Redondo
-*Lithobius forficatus* (family Lithobiidae, same family as *Eupolybothrus cavernicolus*): Birgit Lang, based on a drawing by C. L. Koch

Diplopoda: -*Polyxenus lagurus* (family Polyxenidae, same family as *Eudigraphis taiwanensis*): Guillaume Dera
-*Glomeris marginata*: Thomas Hegna
-*Zoosphaerium* (order Sphaerotheriida, same order and morphology as *Cyliosoma*): Ferran Sayol
-Julida (order comprising *Narceus americanus*): Gemma Martínez-Redondo

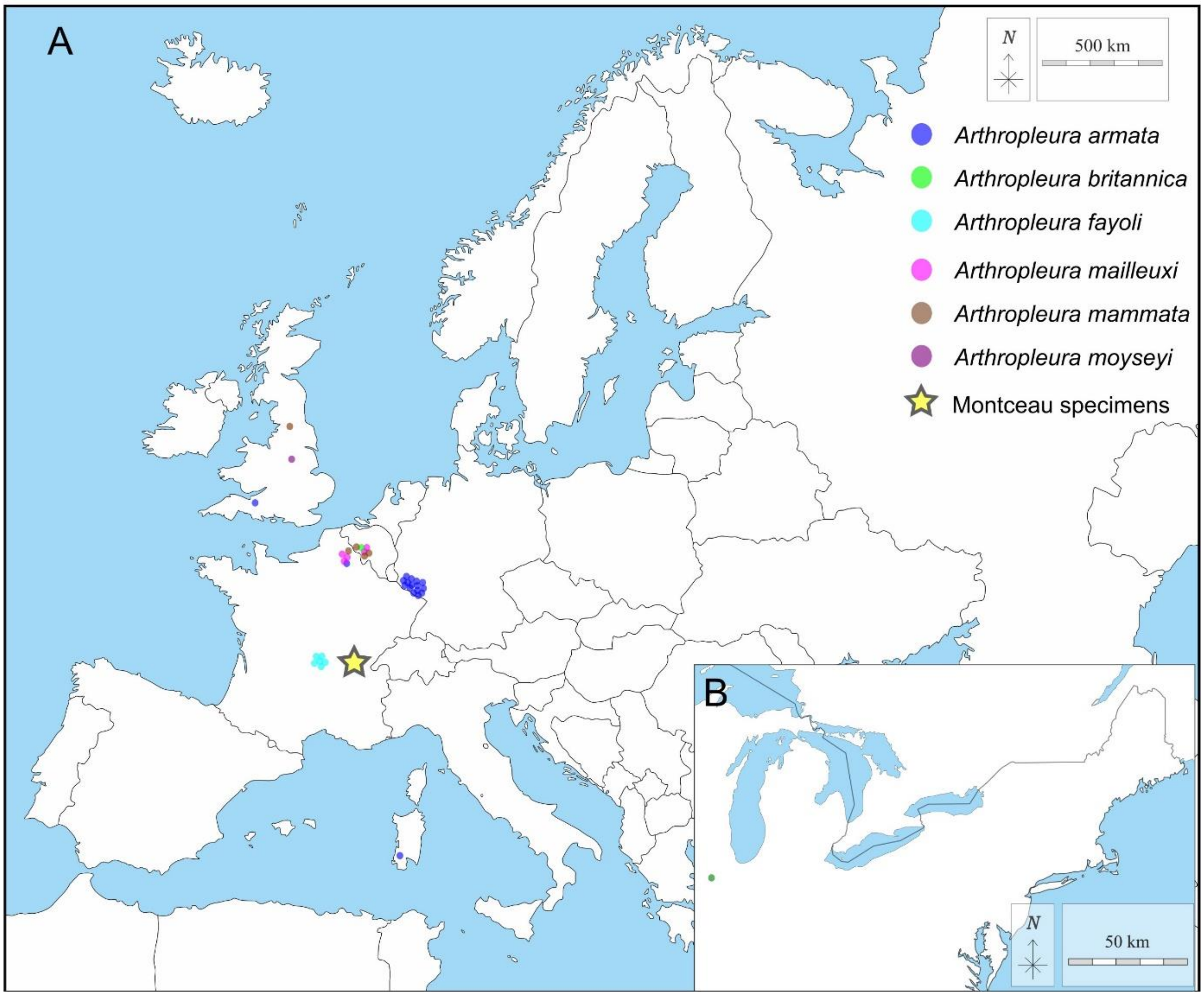

Figure S1. Occurrences of the different *Arthropleura* species. (**A**) *Arthropleura* species occurrences in Europe. (**B**) Rough position of Mazon Creek locality (dark green) where *Arthropleura cristata* is found. Number of occurrences for European species: *A. armata* = 17; *A. britannica* = one; *A. fayoli* = six; *A. mailleuxi* = five; *A. mammata* = five; *A. moyseyi* = one; Montceau specimens = seven. Vectorised maps from © d-maps.com: A from https://d-maps.com/carte.php?num_car=2232&lang=fr ; B from https://d-maps.com/carte.php?num_car=1816&lang=fr.

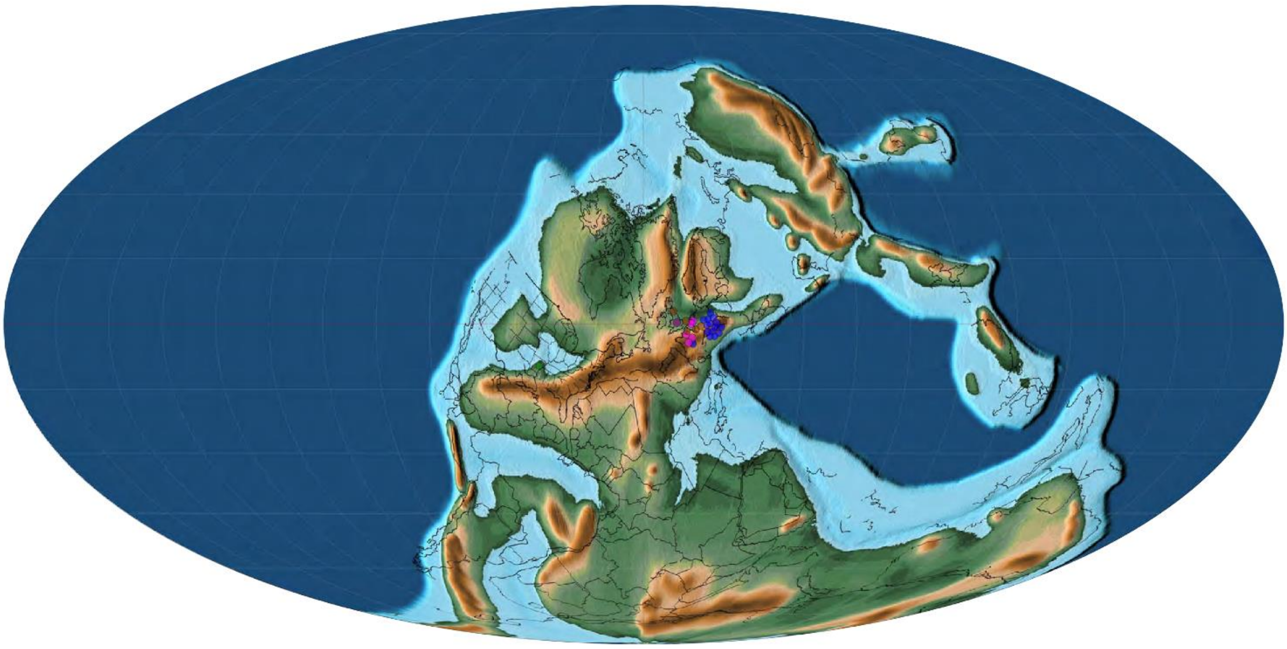

Figure S2. Paleo-localisations of *Arthropleura* occurrences for Early Pennsylvanian, Bashkirian. Colour code for *Arthropleura* species same as Fig. S1. Paleomap from Scotese 2014 (*57*), age 314.9 Ma.

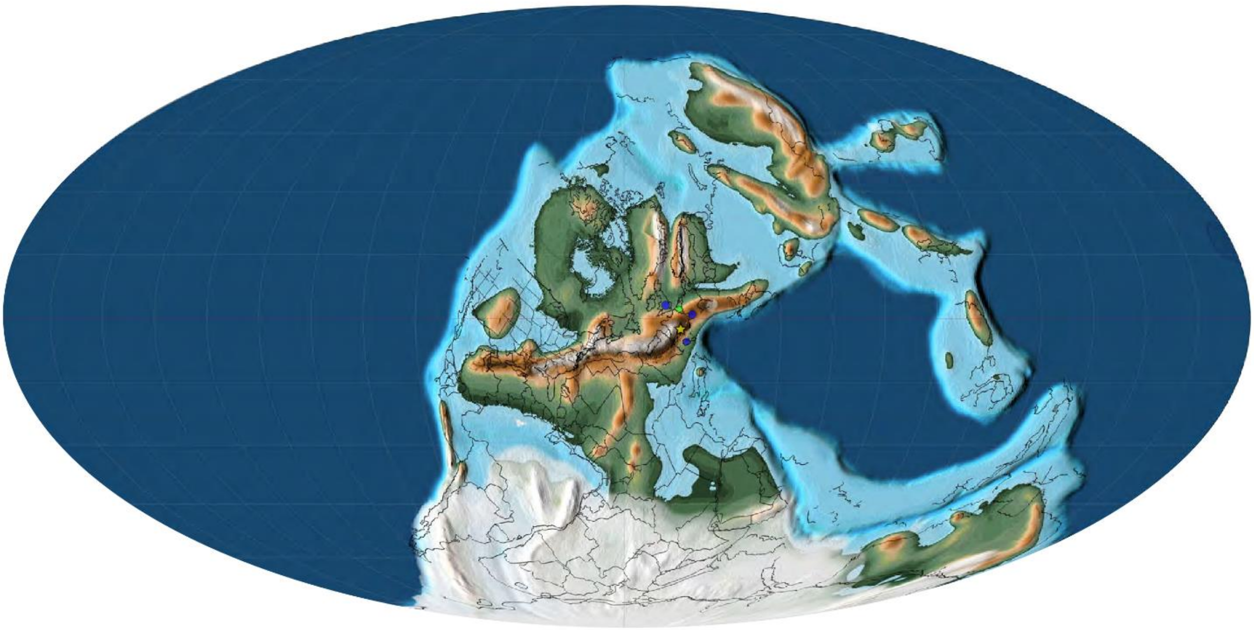

Figure S3. Paleo-localisations of *Arthropleura* occurrences for Late Pennsylvanian, Kasimovian. Colour code for *Arthropleura* species same as Fig. S1. Paleomap from Scotese 2014 (*57*) , age 305.3 Ma.

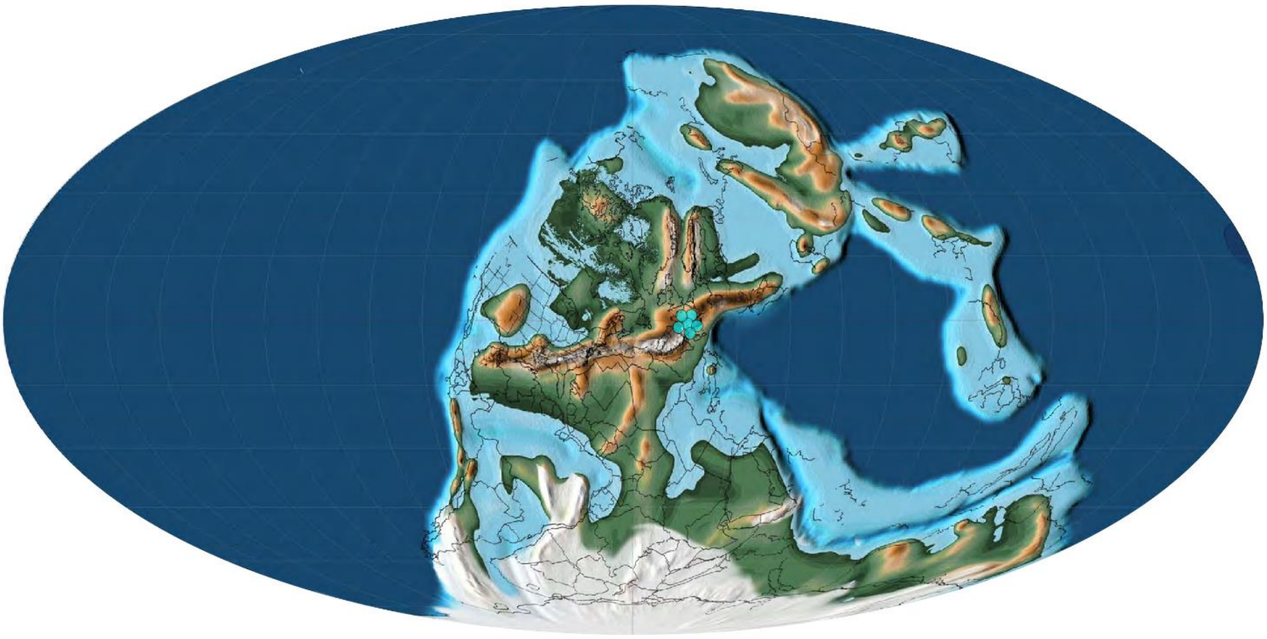

Figure S4. Paleo-localisations of *Arthropleura* occurrences for Late Pennsylvanian, Gzhelian. Colour code for *Arthropleura* species same as Fig. S1. Paleomap from Scotese 2014 (*57*), age 301.2 Ma.

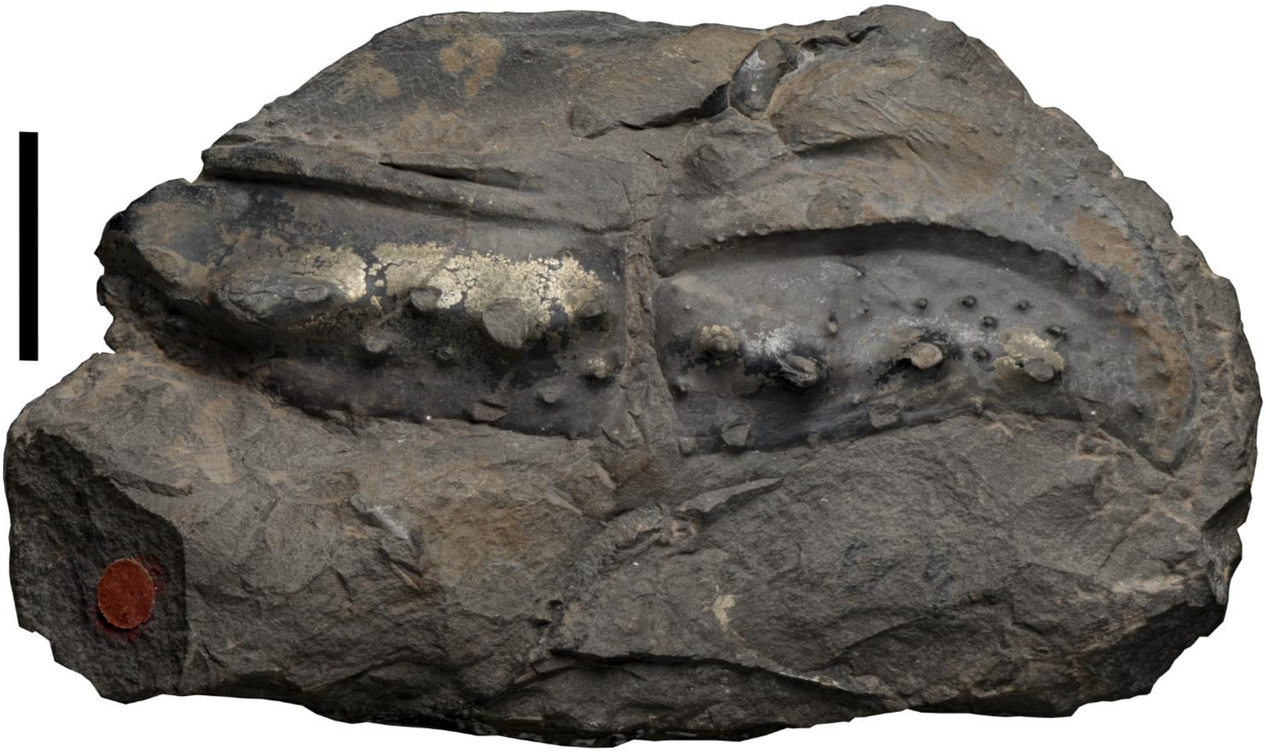

Figure S5. ***Arthropleura armata*.** Holotype MB.A.0614.a-b. Deposited in Museum für Naturkunde, Berlin. Scale bar, 2 cm.

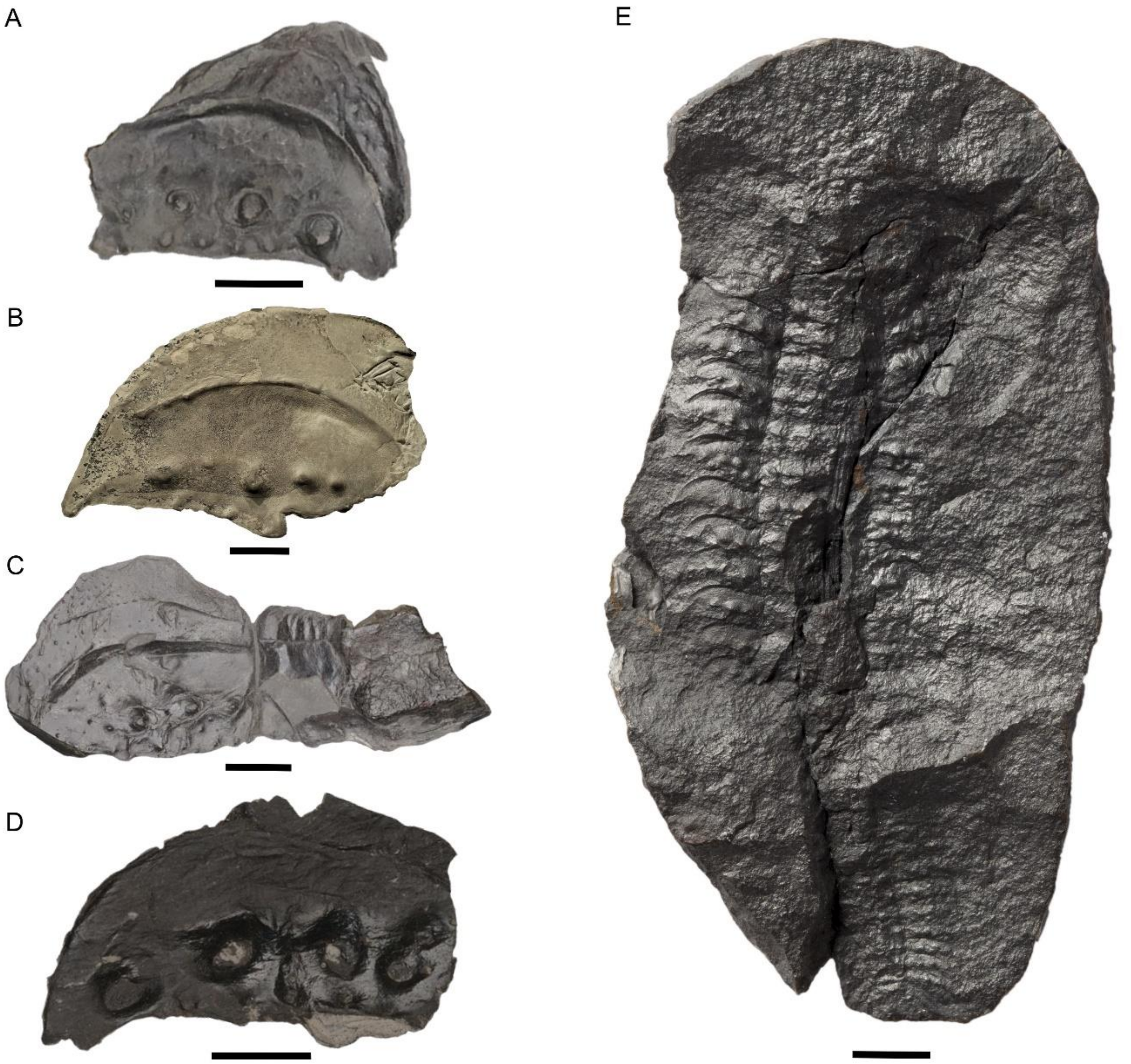

Figure S6. Photos of different *Arthropleura* species. (**A**) *Arthropleura britannica*, specimen IRSNB a 07737. (**B**) *Arthropleura fayoli,* syntype specimen MNHN.F.A31045. (**C**) *Arthropleura mailleuxi*, specimen IRSNB a 07739. (**D**) *Arthropleura mammata*, specimen IRSNB a 07734. (**E**) *Arthropleura moyseyi*, holotype specimen GSM30211Cprt. Scale bars, 2 cm (C); 1 cm (A, B, D); 500 µm (E). Specimens of *A. britannica, A. mailleuxi*, *A. mammata* are property of the Institut Royal des Sciences Naturelles de Belgique, repository number IRSNB. Specimen of *A. fayoli* is property of the Museum National d'Histoire Naturelle, repository number MNHN.F. Specimen *A. moyseyi* is property of the British Geological Survey, repository number BGS GSM. (E) made by the British Geological Survey, CC BY-NC-SA 3.0 (3d-fossils.ac.uk).

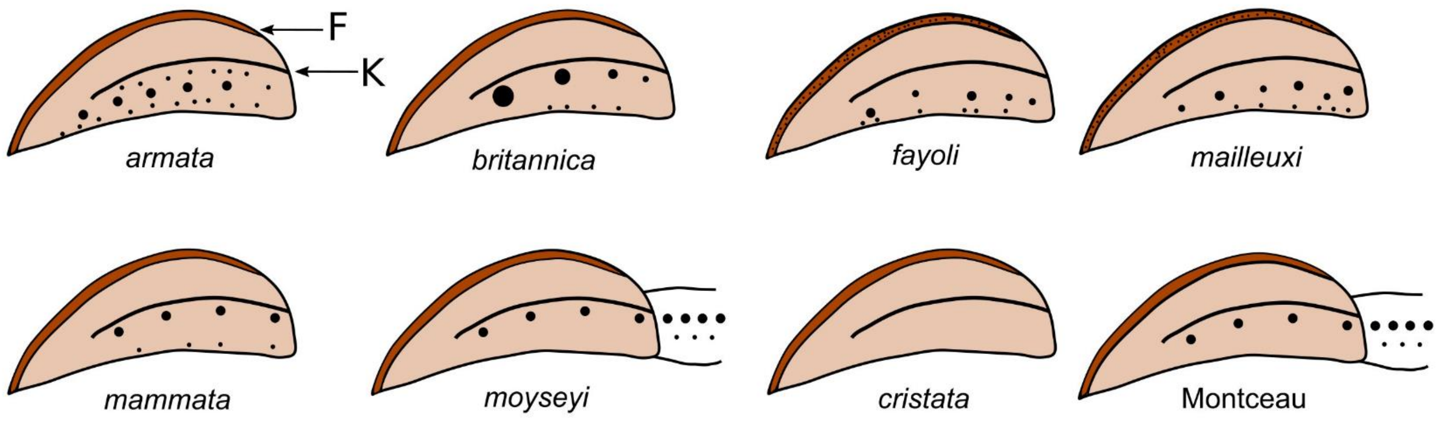

Figure S7. Tubercle patterns of the different *Arthropleura* species. F, furrow; K, keel.

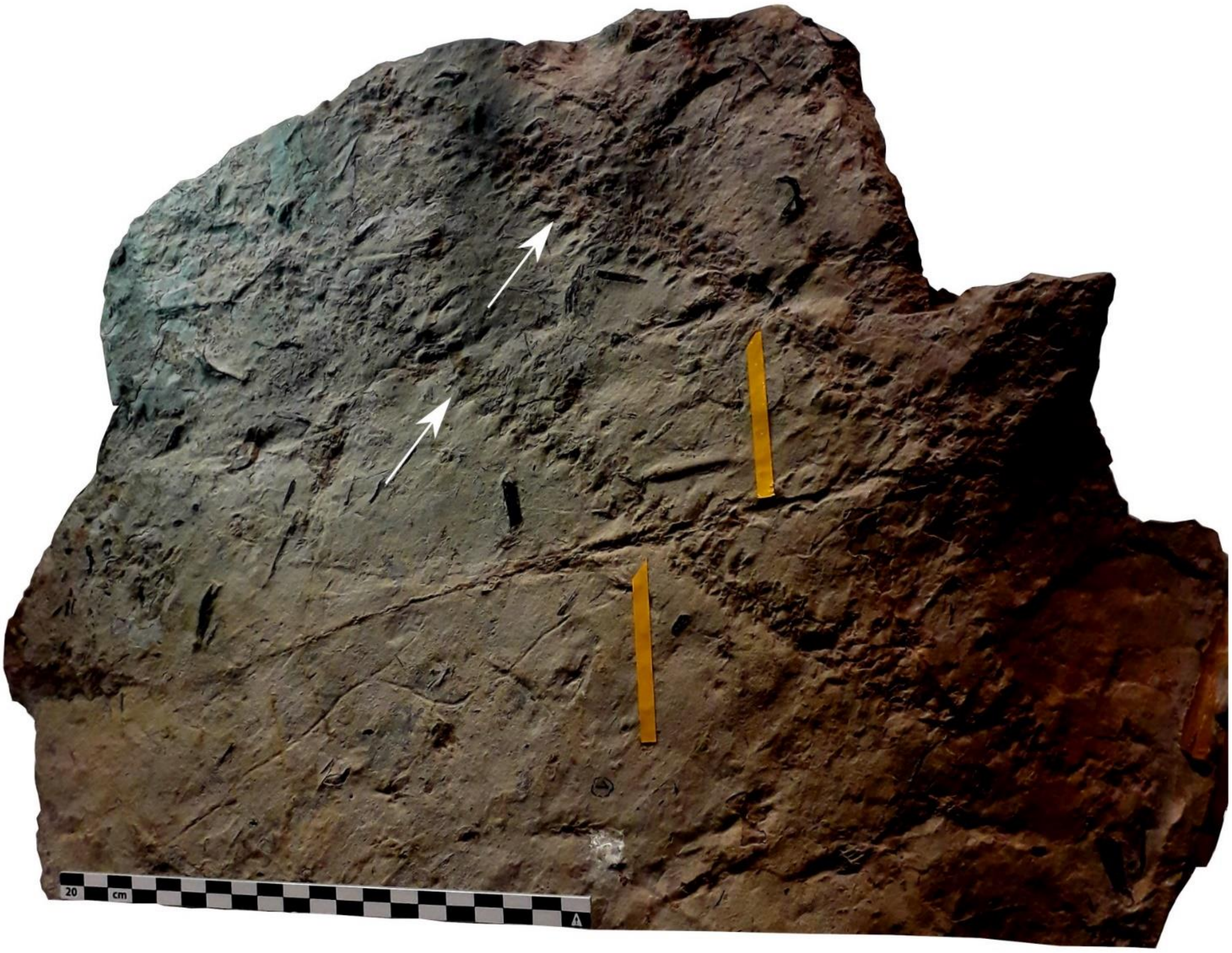

Figure S8. Trackways of *Arthropleura* sp. found in Montceau. White arrows indicate the trackways.

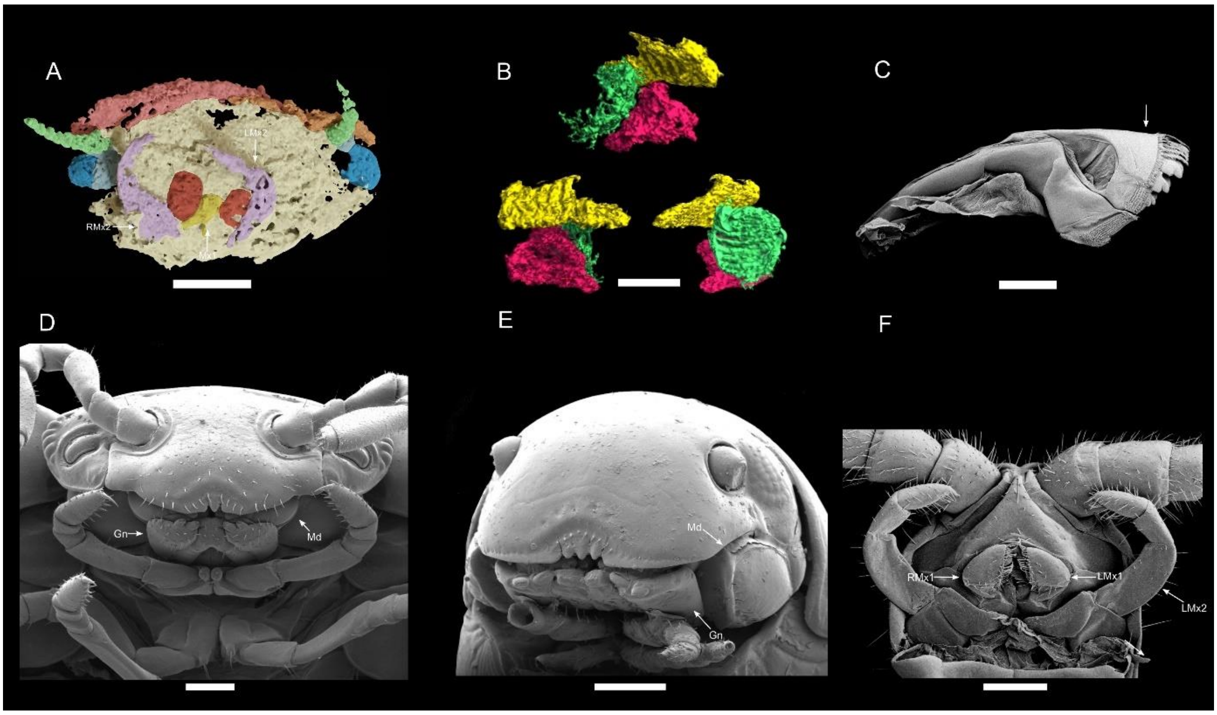

Figure S9. Comparisons of feeding apparatus between *Arthropleura* sp. and extant millipedes and centipedes. (**A-B**) *Arthropleura* sp., specimen MNHN.F.SOT002123. (**A**) ventral view. (**B**) mandible elements, top: ventral view, bottom left: frontal view, bottom right: left lateral view. (**C**) mandible of *Thereuopoda longicornis* (Chilopoda, Scutigeromorpha). (**D-E**) feeding apparatus of extant millipedes. (**D**) *Glomeris marginata* (Diplopoda, Glomerida). (**E**) extant Juliformia (Diplopoda, Juliformia). (**F**) maxillae of *Paralamyctes grayi* (Chilopoda, Lithobiomorpha). Gn, gnathochilarium; LMx1, left first maxilla; LMx2, left second maxilla; Md, mandible; Mx1, first maxilla; RMx1, right first maxilla; RMx2, right second maxilla. White arrow in (**C**) indicates the distal part of the mandible. (**C**), (**F**) provided by Gregory D. Edgecombe. (**D**), (**E**) provided by Mickaël Lhéritier. Scale bars, 1 mm (A); 500 µm (D-E); 400 µm (B, F) and 300 µm (C).

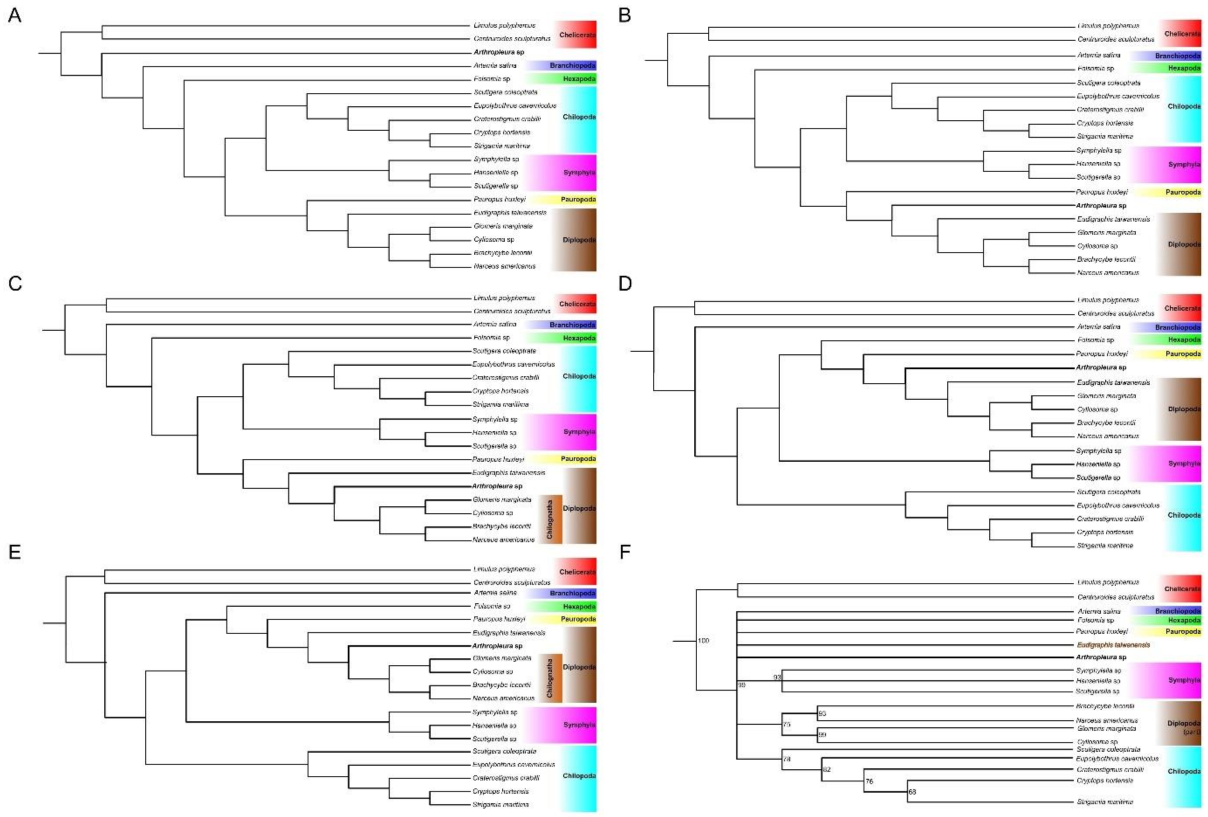

Figure S10. Summary of phylogenetic results obtained for the morphological dataset under maximum parsimony. (**A**) – (**E**)**:** All five MPTs. (**F**): Strict consensus of all most-parsimonious trees (MPTs), with support values (bootstrap frequencies; 1,000 replicates) shown above nodes. The lack of resolution of the strict consensus is partly induced by the unstable position of *Arthropleura* (see Fig. S12 below). Total length = 244 steps; consistency index = 0.865; retention index = 0.787.

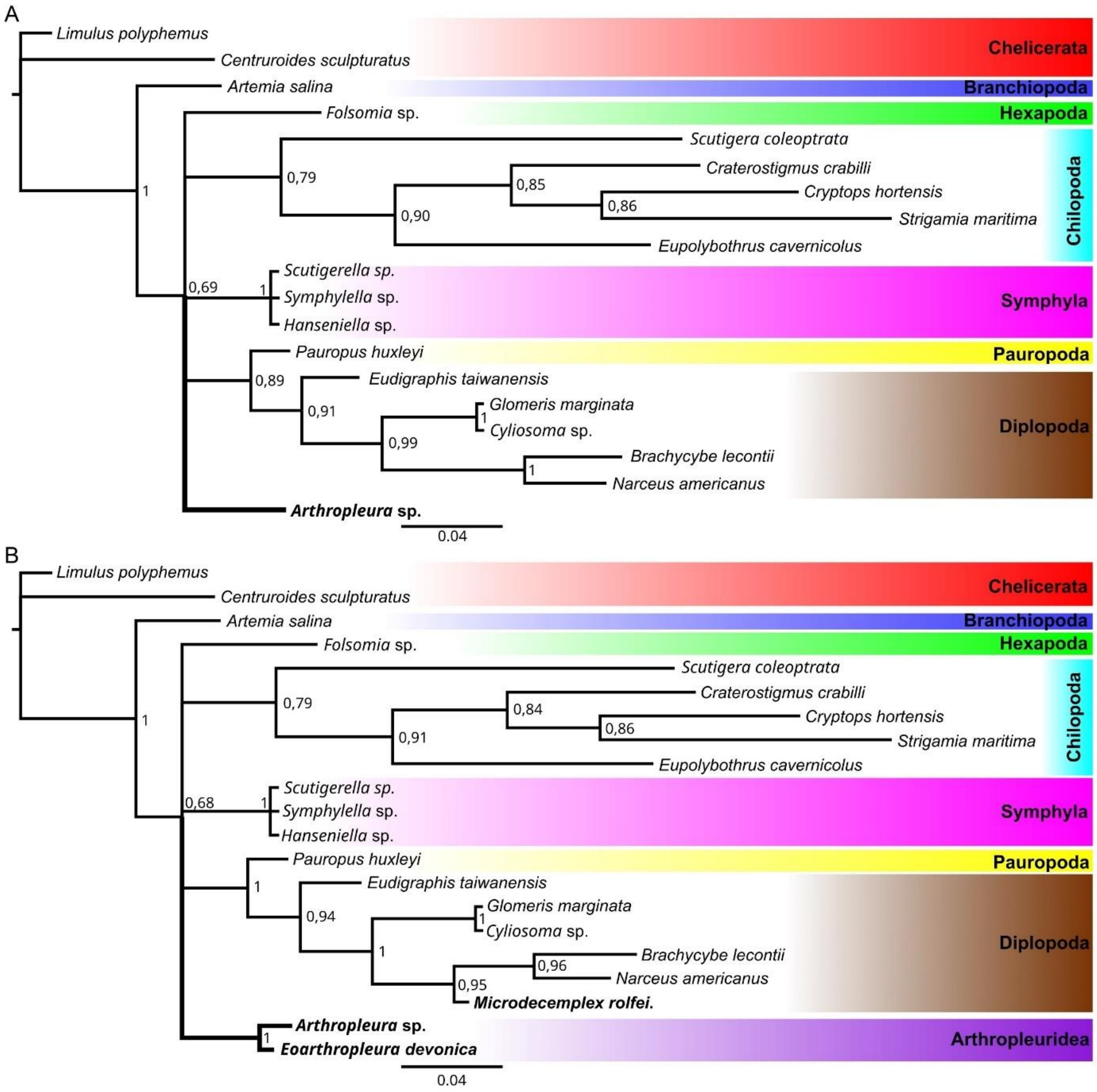

Figure S11. Phylogenetic result obtained for the morphological dataset under Bayesian inference. (A): analysis with only *Arthropleura* sp. **(B)**: analysis with *Arthropleura* sp., *Eoarthropleura devonica* and *Microdecemplex rolfei*. Topology corresponds to a 50% majority-rule consensus, node labels are posterior probabilities (pp). Results are similar to those of MP, both in topology and in the overall weak support for the placement of *Arthropleura* and the backbone of the myriapod tree. Across posterior trees, *Arthropleura* is found with pp > 0.05 as sister group to: A) Hexapoda + Myriapoda (pp = 0.28); B) Branchiopoda (pp = 0.12); C) Pancrustacea + Myriapoda (pp = 0.11); D) Diplopoda (pp = 0.09); E) Diplopoda + Pauropoda (pp = 0.07); F) Pleurostigmophora (i.e., all Chilopoda except for Scutigeromorpha; pp = 0.06); G) Chilopoda (pp = 0.06).

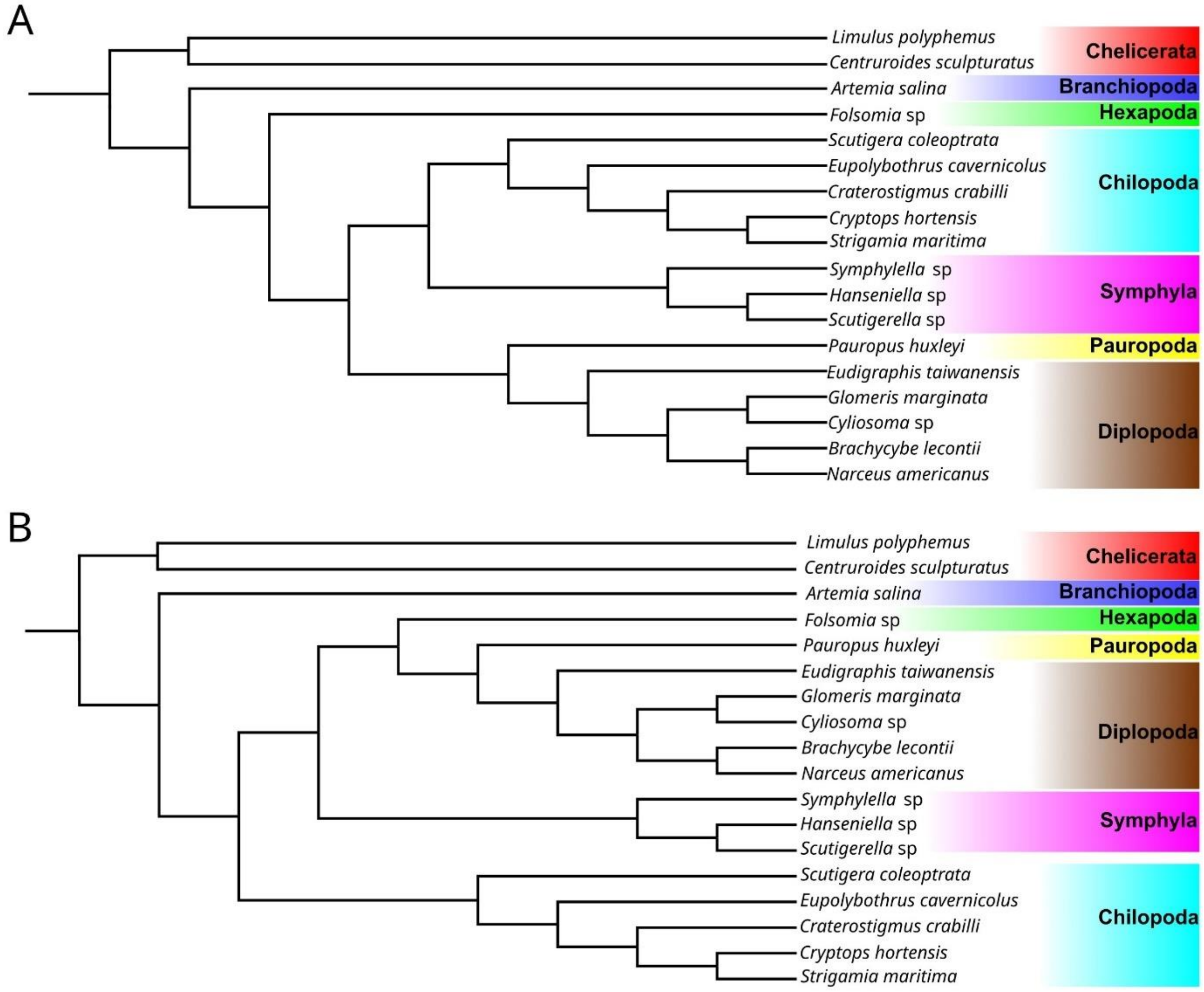

Figure S12. Most parsimonious topologies obtained after the exclusion of *Arthropleura*. (**A**): This topology among myriapods recapitulates previous results (Figs. S11-S13), supporting a split between Chilopoda, Symphyla and Diplopoda + Pauropoda (Dignatha). (**B**): Topology where *Folsomia* is grouped with Pauropoda + Diplopoda because of shared characters on the antennal articles. Total length = 237 steps; consistency index = 0.878; retention index = 0.808.

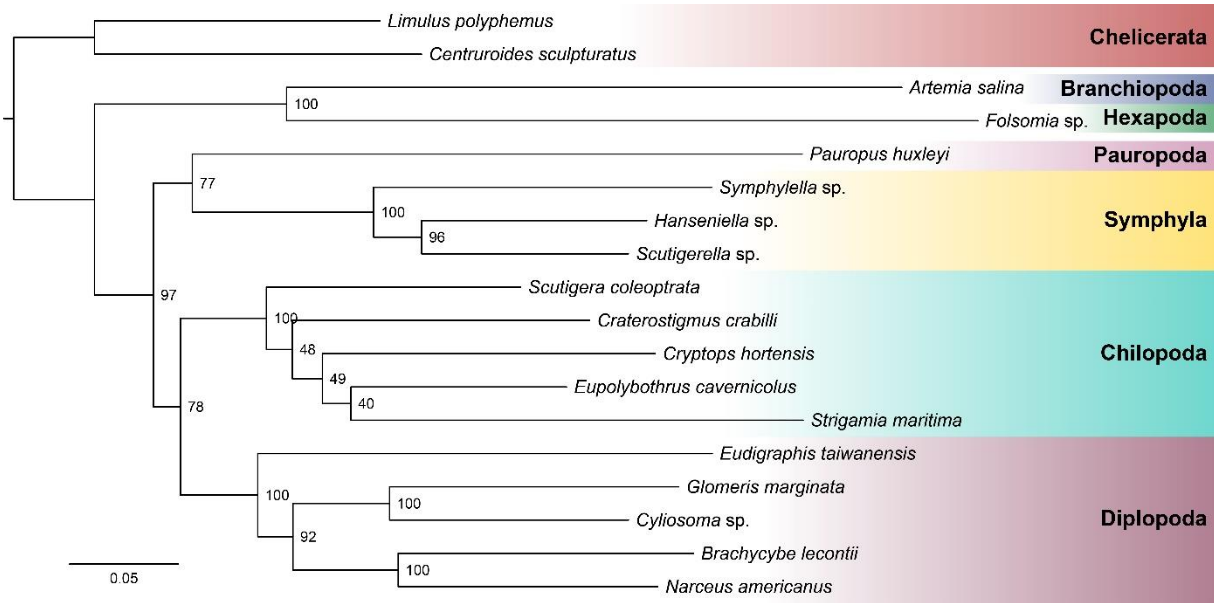

Figure S13. Partitioned maximum likelihood phylogeny derived from the subsampled version of the molecular dataset of Benavides et al. (2023), used also for total-evidence inference (51 high-occupancy loci, 14,570 positions). Node labels correspond to support values derived from 1,000 replicates of ultrafast bootstrap. The topology is identical to that of the full matrix used by Benavides et al., apart from one node in Chilopoda (Lithobiomorpha + Geophilomorpha), and consistent with the monophyly of Edafopoda (Symphyla + Pauropoda) and Pectinopoda (Chilopoda + Diplopoda).

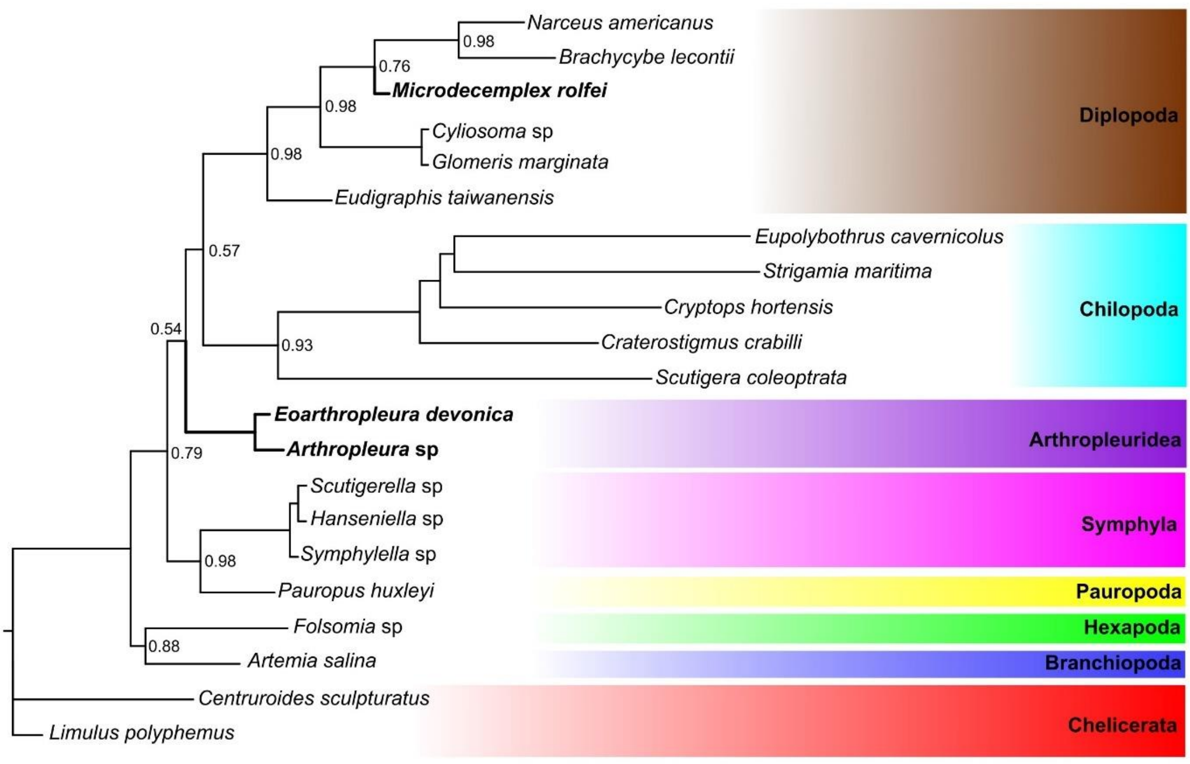

Figure S14. Constrained tree within a molecular backbone topology with all three fossil taxa included. Node values are posterior probabilities, all values >0.99 omitted. Fossil taxa in bold.

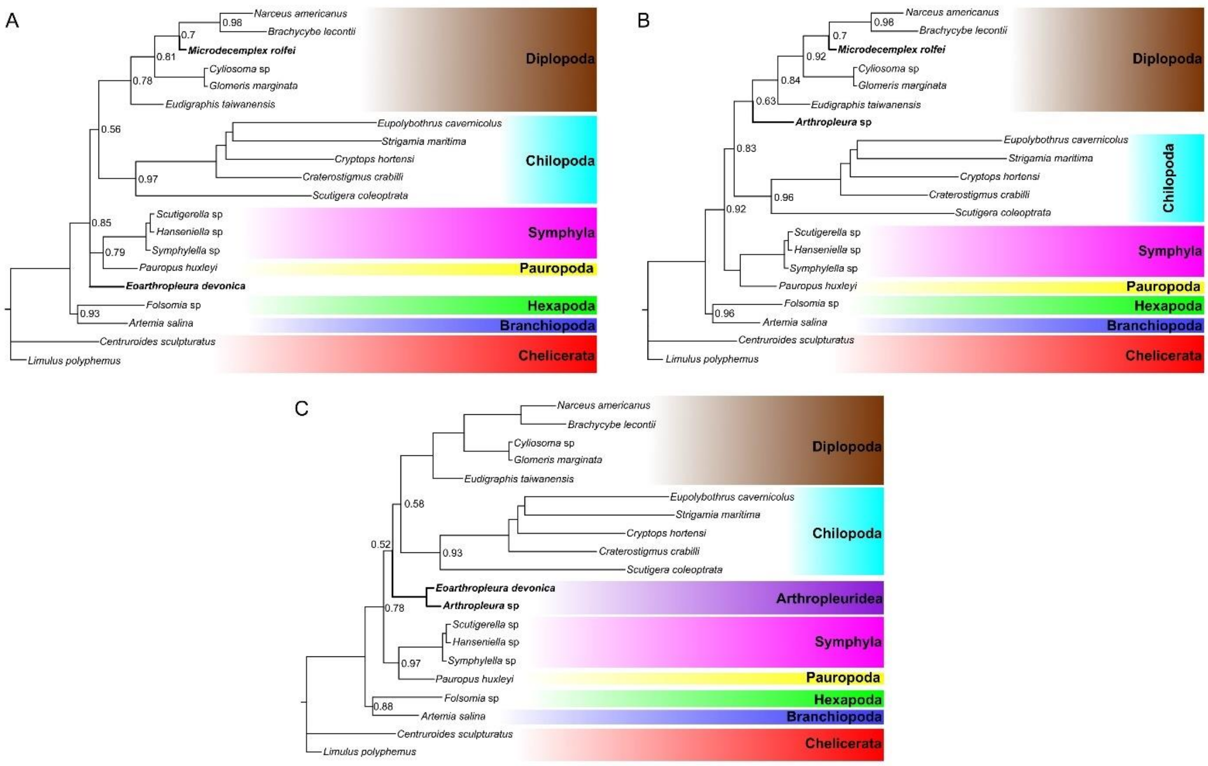

Figure S15. Constrained tree within a molecular backbone topology with one fossil removed. (**A**) without *Arthropleura* sp. (**B)** without *Eoarthropleura devonica*. (**C**) without *Microdecemplex devonica*. Node values are posterior probabilities, all values >0.99 omitted. Fossil taxa in bold.

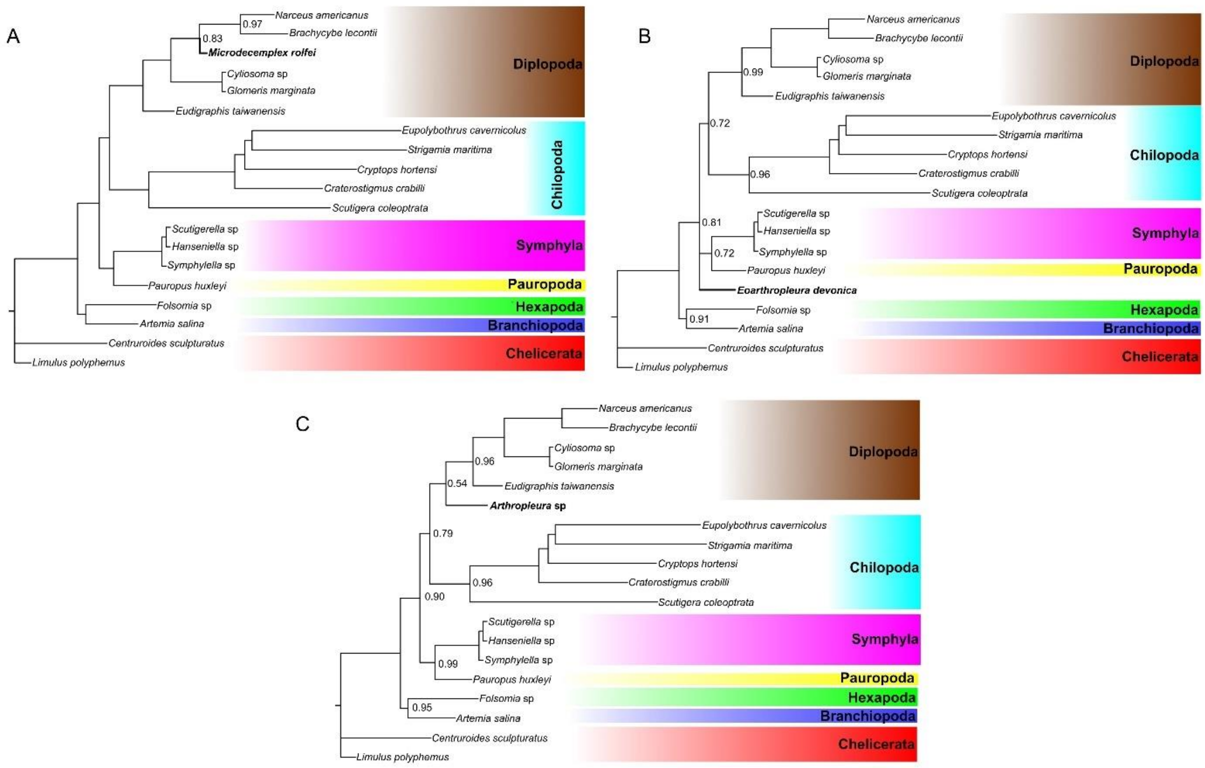

Fig S16. Constrained tree within a molecular backbone topology with two fossil taxa removed. (**A**) without *Arthropleura* sp. and *Eoarthropleura devonica*. (**B**) without *Arthropleura* sp. and *Microdecemplex rolfei*. (**C**) without *Eoarthropleura devonica* and *Microdecemplex rolfei*. Node values are posterior probabilities, all values >0.99 omitted. Fossil taxa in bold.

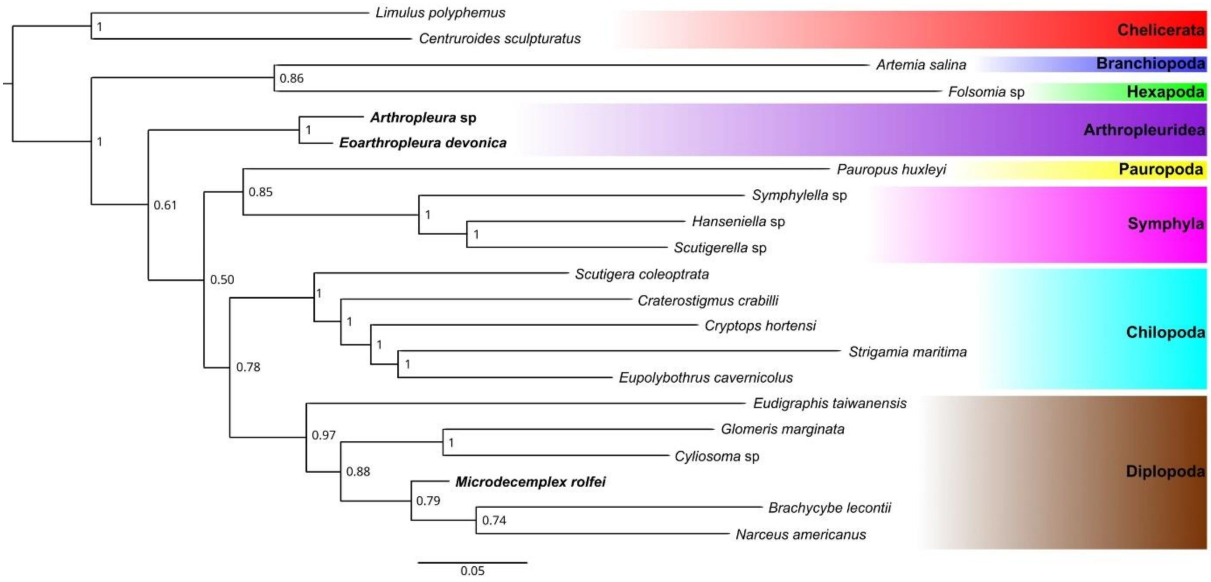

Fig S17. Total evidence partitioned Bayesian phylogeny with *Eoarthropleura devonica* and *Microdecemplex rolfei*. Node numbers are posterior probabilities.

	MNHN.F.SOT.002118	MNHN.F.SOT.002122	MNHN.F.SOT.002123
Number of segments	20	19	24
Body length	23.1	36.6	39.8
Collum length	1.5	0.9	1.2
Collum width	n/a	3.5	4.7
Telson length	2.4	n/a	1.5
Telson width	1.4	n/a	0.6
Sternite length	n/a	n/a	1.9
Sternite width	n/a	n/a	4.2
Paratergite Length	1.9	1.8	1.6
Paratergite width	8.75	5.2	2.7
Syntergite length	1.8	2.7	1.6
Syntergite Width	4.9	4.2	4.4
Head length	4.4	3.5	*3.4*
Head width	n/a	3.9	*5.6*
Leg length	2.2	3.4	5.7
Gut length	n/a	n/a	6

Table S1. **General measurements**. Sternite, syntergite and paratergite length and width are a mean of each available segment measure for each specimen. See further tables for details on syntergites and paratergites. Values in millimetres

Syntergite number	MNHN.F.SOT.002118	MNHN.F.SOT.002122	MNHN.F.SOT.002123
2	1.7	1.3	2.7
3	2.6	1.6	1.8
4	n/a	2.4	1.8
5	n/a	2	1.9
6	n/a	3	2
7	n/a	2.8	1.4
8	n/a	2.6	1.6
9	n/a	2	1.6
10	2.4	1.7	1.9
11	2.3	2	1.1
12	2.8	2.2	1.1
13	1.9	1.4	1.6
14	1.6	1.2	1.3
15	1.5	1.2	1.1
16	1.9	1.6	0.9
17	1.6	1.5	0.9
18	1.4	1.3	0.8
19	0.9	1.9	0.7
20	0.7		0.6
21	1.4		0.5
22			1
23			0.6

Table S2. **Syntergite lengths.** Collum and telson lengths on Table S1. Values in millimetres.

Syntergite number	MNHN.F.SOT.002118	MNHN.F.SOT.002122	MNHN.F.SOT.002123
2	n/a	3.6	3.3
3	n/a	3.8	3.6
4	n/a	4.1	5.1
5	n/a	4.4	5.5
6	n/a	4.7	5.5
7	n/a	4.5	5.6
8	n/a	4.5	5.6
9	n/a	4.8	5
10	n/a	4.7	4.4
11	5.4	4.5	3.5
12	4.3	4.6	4
13	4.4	4.4	3.5
14	5.1	3.8	2.9
15	5.1	3.5	2.5
16	3.7	3	2.3
17	3.8	2.6	2.1
18	2.4	2.3	1.9
19	1.9	2.5	1.5
20	1.9		1.3
21	1.5		1.3
22			1.2
23			1.3

Table S3. Syntergite widths. Collum and telson widths present in Table S1. Values in millimetres.

Paratergite number	MNHN.F.SOT.002118	MNHN.F.SOT.002122	MNHN.F.SOT.002123
2	n/a	1.5	2.6
3	n/a	1.5	1.6
4	n/a	2.6	2
5	n/a	2.4	1.7
6	n/a	2.9	1.7
7	n/a	2.5	1.2
8	n/a	2.4	2
9	n/a	2.1	2
10	2.3	2.3	1.1
11	1.2	1.9	1.2
12	2.3	2.6	1
13	3.1	1.2	1.2
14	2.4	0.8	1.5
15	1.9	1.4	0.6
16	2.4	1.5	0.9
17	1.7	1.5	0.8
18	1.9	0.6	0.8
19	0.6	1.5	0.6
20	1.3		0.6
21	1.2		0.4
22			0.9
23			1.5

Table S4. Paratergite lengths. Values in millimetres.

Paratergite number	MNHN.F.SOT.002118	MNHN.F.SOT.002122	MNHN.F.SOT.002123
2	n/a	4.9	3.2
3	n/a	5.1	3.4
4	n/a	5.1	3.2
5	n/a	5.8	3
6	n/a	5.8	3.6
7	n/a	5.7	3.2
8	n/a	4.3	n/a
9	n/a	5.8	2.8
10	9.9	5.6	3.7
11	9.6	5	2.8
12	9.6	5	2.7
13	8.4	4.4	3.1
14	8	2.2	2.8
15	7	3.3	2.4
16	5.8	3.7	2.3
17	3.1	3.6	2.4
18	3.3	3.7	2.2
19	4	3.7	2.4
20	4.7		2.3
21	4.5		1.9
22			1.9
23			1.7

Table S5. Paratergite widths. Values in millimetres.

	MNHN.F.SOT.002118	MNHN.F.SOT.002123	Antenna segment number	length
Left Antenna length	2.7	2.3	1	0.8
Left Antenna width	0.3	0.3	2	0.3
Right Antenna length	n/a	4	3	0.2
Right Antenna width	n/a	0.5	4	0.4
Left Maxilla 1 length	n/a	1.2	5	0.3
Left Maxilla 1 width	n/a	0.4	6	0.3
Right Maxilla 1 length	n/a	1.3	7	0.4
Right Maxilla 1 width	n/a	1		
Left Maxilla 2 length	n/a	2.4		
Left Maxilla 2 Width	n/a	0.7		
Right Maxilla 2 length	n/a	4		
Right Maxilla 2 Width	n/a	1.1		
Left Mandible length	n/a	2.7		
Left Mandible width	n/a	0.9		
Right Mandible length	n/a	4.1		
Right Mandible width	n/a	0.7		
Left Stalk length	0.5	0.8		
Right Stalk length	n/a	1.1		
Left Ocular field length	0.5	0.3		
Left Ocular field width	1.3	0.5		
Left Ocular field thickness	0.3	0.4		
Right Ocular field length	n/a	0.5		
Right Ocular field width	n/a	0.4		
Right Ocular field thickness	n/a	0.2		

Table S6. Measurements on head. Antennal length is made from the sum of each segment length. Values in millimetres.

Leg segment length	MNHN.F.SOT.002118	MNHN.F.SOT.002122	MNHN.F.SOT.002123
coxa		0.4	1.01
prefemur	0.3	0.2	1.03
femur	0.6	0.3	0.75
post-femur	0.3	0.5	1.16
tibia	0.6	1	0.56
tarsus 1	0.2	0.7	0.38
tarsus 2	0.1	0.3	0.46
claw	0.1		0.3
Total	**2.2**	**3.4**	**5.7**

Table S7. Measurements of leg segments. Values in millimetres.

Mandible element	Length	Width
red	0.6	0.5
green	0.4	0.7
yellow	0.9	0.4

Table S8. Measurements of the mandible elements. See Fig. 3 for colour references. Values in millimetres.

Limulus polyphemus
? 2 - 0 0 0 0 0 1 - - - - - - - - 0 0 0
0 ? 0 - 0 ? 1 0 ? ? ? 0 0 0 0 0 ? ? ? ?
? ? ? ? 0 0 0 - - - - - - - - 0 - - - -
- - - 0 ? ? ? ? ? ? ? ? ? ? ? ? 0 - - -
- - - ? ? ? ? ? ? 1 ? 0 0 0 0 0 ? ? 0 0
- ? ? ? ? ? ? ? ? ? ? 0 0 ? 0 ? ? ? ? 0
0 0 ? 0 ? ? ? ? 0 0 ? ? - - - - - - - -
0 ? ? 0 0 0 0 - - ? ? ? 0 - - - - ? - -
- 0 0 0 - - 0 - 0 0 0 0 0 0 0 0 ? ? ? 0
0 0 1 0 0 0 0 - 4 0

Centruroides sculpturatus
? 2 - 0 ? 0 0 0 1 - - - - - - - - 0 0 1
? ? 0 - 0 ? 4 ? ? ? ? 1 0 1 0 0 ? ? ? ?
? ? ? ? 1 0 0 - - - - - - - - 0 - - - -
- - - 0 ? ? ? ? ? ? ? ? ? ? ? ? 0 - - -
- - - ? ? ? ? ? ? 0 ? 0 0 0 0 0 ? ? 0 0
- ? ? ? ? ? ? ? ? ? ? 0 0 ? 0 ? ? ? ? 0
1 0 ? 0 ? ? ? ? 0 1 ? ? - - - - - - - -
? ? ? 0 1 0 0 - - ? ? ? 0 - - - - ? - -
- 0 0 0 - - 0 ? 0 0 0 0 0 0 0 0 ? ? ? 0
0 1 1 1 0 0 0 - 4 0

Artemia salina
? 0 ? ? 0 0 1 0 0 ? ? ? 0 0 0 0 0 - - -
? ? 0 - 0 ? 1 0 ? ? ? 0 0 0 0 0 ? ? ? ?
? ? ? ? 0 0 1 0 0 ? - ? ? ? - 1 0 - ? ?
? ? ? 0 ? 0 ? ? ? ? ? ? ? ? ? ? 0 - - -
- - - ? ? ? ? ? ? 0 ? ? 0 0 0 0 ? ? 0 0
- ? ? ? ? ? ? ? ? ? ? 0 0 ? 0 ? ? ? ? 0
0 1 ? 0 ? ? ? ? 0 0 ? ? - - - - - - - -
? ? ? ? 0 0 0 - - ? ? ? 0 - - - - ? - -
- 0 0 0 - - 0 - 0 ? ? 0 0 0 0 0 ? ? ? 0
0 0 0 - 1 0 0 0 2 0

Folsomia sp.
0 2 - 0 0 0 0 0 0 1 2 0 0 0 0 0 0 - - -
0 ? 1 0 1 ? 3 - - - - 0 1 0 0 1 ? ? ? ?
? ? ? ? 0 0 1 0 0 0 - 0 ? 0 - 1 0 - ? ?
? ? - 0 0 0 ? ? ? ? ? ? ? ? ? ? 0 - - -
- - - 0 ? ? ? ? ? 0 ? 0 1 0 0 0 ? 0 0 0
- ? ? ? ? ? ? ? ? ? ? 2 0 ? 0 ? 0 0 ? 0
0 0 ? 0 ? ? ? ? 1 0 ? ? - - - - - - - -
? ? ? 0 0 0 0 - - ? ? ? 0 - - - - 0 - -
- 0 0 0 - - 0 ? ? ? ? ? ? ? ? ? ? ? ? ?
? 0 0 - 0 0 0 0 1 0

Pauropus huxleyi
- 0 0 0 0 1 0 0 0 1 2 0 1 0 0 1 0 - - -
0 0 1 0 0 0 3 - - - - - 1 0 0 0 ? 0 0 0
? ? ? ? 0 0 1 0 ? 0 - ? ? 0 - 1 1 0 ? -
- - - 0 0 1 - - - - - - - - - ? 0 - - -
- - - 0 0 0 0 0 0 0 ? 0 0 0 0 0 ? 0 0 0
- ? 0 0 0 0 0 0 0 0 0 0 0 0 0 1 0 0 0 0
0 0 ? 0 - 0 ? 0 0 0 ? ? - - - - - - - -
? ? 1 0 0 1 0 - - ? ? ? 0 - - - - 1 - -
- 0 0 0 - - 0 0 ? 0 0 0 0 0 0 0 0 0 - 0
0 0 0 - 0 0 0 0 3 0

Hanseniella sp.
0 0 ? ? 0 0 0 0 0 0 0 0 0 0 0 0 0 - - -
0 0 1 0 0 ? 3 - - - - - 1 0 0 0 0 0 0 0
? ? ? ? 0 0 1 0 ? 0 - 0 ? 0 - 1 0 - ? -
- - - 0 0 0 - - - - - - - - - ? 0 - - -
- - - 0 0 0 0 0 0 0 ? 0 0 0 0 0 ? 0 0 0
- ? 0 0 0 0 0 0 0 0 0 1 0 0 0 0 0 0 0 0
0 0 ? 0 - 0 ? 0 0 0 ? ? - - - - - - - -
? ? 1 0 0 1 0 - - ? ? ? 0 - - - - 3 - -
- 0 0 0 - - 0 0 ? ? ? ? ? ? 1 0 0 0 0 0
1 0 0 - 0 0 0 0 3 0

Scutigerella sp.
? 0 ? ? 0 ? 0 0 0 0 0 0 0 0 0 0 0 - - -
0 0 1 0 0 ? 3 - - - - - 1 0 0 0 0 0 0 0
? ? ? ? 0 0 1 0 ? 0 - 0 ? 0 - 1 0 - ? -
- - - 0 0 0 - - - - - - - - - ? 0 - - -

- - - 0 0 0 0 0 0 0 ? 0 0 0 0 0 ? 0 0 0
- ? 0 0 0 0 0 0 0 0 0 1 0 0 0 0 0 0 0 0
0 0 ? 0 - 0 ? 0 0 0 ? ? - - - - - - - -
? ? ? 0 0 1 0 - - ? ? ? 0 - - - - 3 - -
- 0 0 0 - - 0 0 1 0 0 0 0 0 1 0 0 0 0 0
1 0 0 - 0 0 0 0 3 0

Symphylella sp.
? 0 ? ? ? ? 0 0 0 0 0 0 0 0 0 0 0 - - -
0 0 1 0 0 ? 3 - - - - - 1 0 0 0 0 0 0 0
? ? ? ? 0 0 1 0 ? 0 - 0 ? 0 - 1 0 - ? -
- - - 0 ? 0 - - - - - - - - - ? 0 - - -
- - - 0 0 0 0 0 0 0 ? 0 0 0 0 0 ? 0 0 0
- ? 0 0 0 0 0 0 0 0 0 1 0 0 0 0 0 0 0 0
0 0 ? 0 - 0 ? 0 0 0 ? ? - - - - - - - -
? ? ? 0 0 ? 0 - - ? ? ? 0 - - - - 3 - -
- 0 0 0 - - ? ? ? ? ? ? ? ? 1 0 0 0 0 0
1 0 0 - 0 0 0 0 3 0

Scutigera cloleoptrata
1 0 2 0 0 0 0 0 0 0 0 1 0 0 1 0 0 - - -
0 1 1 0 0 1 1 - 0 0 0 - 1 0 0 0 1 0 0 1
0 0 0 0 0 0 1 0 0 1 0 0 0 0 0 1 0 - 0 0
0 1 0 1 1 0 0 0 0 0 1 0 0 0 - 0 1 0 0 0
0 0 0 0 1 0 0 1 0 0 0 0 0 0 0 1 0 0 0 1
0 0 0 0 0 0 1 1 0 0 0 0 1 0 0 1 1 0 1 1
0 0 0 0 0 0 0 0 0 0 0 0 1 - - 0 0 - - -
0 0 0 0 0 1 0 - - 0 0 - 1 1 1 0 - 0 2 0
1 0 0 0 - - 0 0 1 0 0 1 0 0 0 0 0 0 0 0
0 0 0 - 0 0 0 1 4 0

Craterostigmus crabilli
? 0 3 1 0 ? 0 0 0 1 0 0 0 0 0 0 0 - - -
1 2 1 0 0 0 2 - 1 1 0 - 1 0 0 0 3 0 0 0
? 0 0 0 0 0 1 0 1 0 ? 0 0 0 1 1 0 - 0 1
0 0 0 0 2 0 1 0 0 1 0 0 1 1 1 1 1 1 1 0
2 1 1 1 0 0 0 0 0 0 0 0 0 0 0 1 0 0 0 0
1 1 0 0 0 0 0 0 0 0 0 0 0 0 0 0 0 1 0 0
0 0 0 1 1 0 0 1 0 0 ? 1 1 - - 0 0 - - -
? ? ? 0 0 1 0 - - ? ? ? 0 - - - - 0 1 1
- 1 1 1 1 2 1 ? ? ? ? ? ? ? 0 0 0 0 0 0
0 0 0 - 0 0 0 1 4 0

Cryptops hortensis
1 2 - 1 1 0 0 0 0 1 0 0 0 0 0 - 0 - - -
1 0 1 0 0 0 3 - - - - - 0 0 0 0 1 0 0 0
0 0 0 1 0 0 1 1 0 0 0 0 1 0 0 1 0 - 0 0
1 0 0 0 2 0 1 - 0 1 0 0 2 1 2 0 1 1 2 0
2 1 1 1 0 0 1 0 1 0 1 0 0 0 0 1 1 0 0 0
0 2 1 1 0 0 0 0 0 0 1 0 0 0 0 0 0 0 0 0
0 0 0 0 1 0 1 0 0 0 0 1 1 - - 1 0 - - -
1 1 ? 0 0 1 0 - - ? ? ? 0 - - - - 0 - 1
- 0 0 1 1 1 1 1 ? 0 0 1 0 0 0 0 0 0 0 0
0 0 0 - 0 0 0 1 4 0

Srigamia maritima
1 2 - 2 1 0 0 0 0 1 1 0 0 0 0 - 1 - - -
1 0 1 0 0 0 3 - - - - - 0 0 0 0 3 0 0 0
1 1 0 0 0 0 1 0 0 0 1 1 - - - 1 0 - 1 0
0 0 0 0 2 0 1 1 1 1 0 0 0 1 0 0 1 1 0 0
2 1 1 1 0 1 2 0 0 0 2 1 0 0 0 0 0 0 0 0
- 2 0 0 0 0 0 0 0 1 0 0 0 0 0 0 0 0 0 0
0 0 0 0 1 1 0 0 0 0 1 1 0 - - 1 1 - - -
0 0 ? 0 0 1 0 - - 1 1 1 1 0 2 0 - 0 0 1
- 0 1 1 1 1 1 ? 0 0 0 1 0 0 0 0 0 0 0 0
0 0 0 - 0 0 0 1 4 0

Eupolybothrus cavernicolus
? 0 ? 0 0 ? 0 0 0 0 0 0 0 0 0 0 0 - - -
1 2 1 0 0 0 0 0 1 1 1 - 1 0 0 0 1 1 1 0
0 0 1 0 0 0 1 0 1 0 0 0 1 0 0 1 0 - 0 0
0 2 1 0 ? 0 1 0 0 1 0 1 0 1 1 1 1 0 0 1
1 0 1 1 0 0 0 0 0 0 0 0 0 0 0 1 0 0 0 0
0 0 0 0 1 1 0 0 0 0 0 0 0 1 1 1 0 0 0 0
0 0 0 0 0 0 0 0 0 0 0 0 1 - - 0 0 - - -
? 0 ? 0 0 1 0 - - ? ? - 1 1 0 1 1 0 2 1
- 0 2 1 0 1 1 ? ? ? ? ? ? ? 0 0 0 0 0 0

0 0 0 - 0 0 0 1 4 0

Eudigraphis taiwanensis
- 0 0 0 0 1 0 0 0 1 2 0 0 1 0 0 0 - - -
0 0 1 0 0 ? 1 0 0 0 0 - 1 0 0 0 0 0 0 0
? ? ? ? 0 0 1 0 0 0 0 0 ? 0 - 1 1 0 ? -
- - - 0 ? 1 - - - - - - - - - - 0 - - -
- - - 0 0 0 0 0 0 0 ? 0 0 1 0 0 ? 0 0 0
- 0 0 0 ? 0 0 0 0 0 0 0 0 0 0 1 0 0 0 0
0 0 1 0 - 0 ? 0 0 0 ? 0 - 0 0 0 0 0 0 -
0 ? ? 0 0 1 0 - - ? ? ? 0 - - - - 1 - -
- 0 0 0 - - 0 ? ? ? ? ? ? ? 0 0 0 0 0 0
0 0 0 - 0 1 0 1 7 0

Glomeris marginata
- 0 0 0 0 1 0 0 0 1 2 0 0 1 0 0 0 - - -
0 0 1 0 0 0 0 0 0 0 0 - 1 0 1 0 1 0 0 0
? ? ? ? 0 0 1 0 0 0 0 0 ? 1 - 1 2 1 ? -
- - - 0 0 1 - - - - - - - - - ? 0 - - -
- - - 0 0 0 0 0 0 0 ? 0 0 1 0 0 ? 1 0 0
- 0 0 0 ? 0 0 0 0 0 0 0 0 0 0 0 0 0 0 0
0 0 0 0 - ? ? 0 0 0 ? 0 - 1 0 1 0 0 1 1
0 ? 1 1 0 1 0 - 0 ? ? ? 0 - - - - 1 - -
- 0 0 0 - - 0 0 0 1 - 0 0 0 0 0 0 0 0 1
0 0 0 - 0 0 0 1 3 0

Cyliosoma sp.
- 0 ? 0 0 ? 0 0 0 1 2 0 0 1 0 0 0 - - -
0 0 1 0 0 0 0 0 ? ? ? - 1 0 1 0 1 0 0 0
? ? ? ? 0 0 1 0 0 0 0 0 ? 1 - 1 2 1 ? -
- - - 0 ? 1 - - - - - - - - - - 0 - - -
- - - 0 0 0 0 0 0 0 ? 0 0 1 0 0 ? 1 0 0
- 0 0 0 ? 0 0 0 0 0 0 0 0 0 0 0 0 0 0 0
0 0 0 0 - ? ? 0 0 0 ? ? - 1 0 1 0 0 1 1
? ? ? 1 0 1 0 - - ? ? ? 0 - - - - 1 - -
- 0 0 0 - - 0 ? 0 1 - 0 0 0 0 0 0 0 0 1
0 0 0 - 0 0 0 1 3 0

Brachycybe lecontii
- 1 1 1 0 ? 0 0 0 1 2 0 0 1 0 0 0 - - -
0 0 1 0 0 ? 3 - - - - - 0 0 1 0 0 0 ? 0
? ? ? ? 0 0 1 ? 0 0 0 0 ? 1 - 1 2 0 ? -
? ? - 0 ? 1 - - - - - - - - - ? 0 - - -
- - - 0 0 0 0 0 0 0 ? ? 0 1 1 0 ? 0 0 0
- 0 0 0 ? 0 0 0 0 0 0 0 0 0 0 0 0 0 0 0
0 0 1 0 - ? ? 0 0 0 ? ? - 2 1 0 0 0 1 0
? ? ? 0 0 1 1 0 ? ? ? ? 0 - - - - 1 - -
- 0 0 0 - - 0 ? ? ? ? ? ? ? 0 1 0 1 1 0
0 0 0 - 0 0 0 1 4 0

Narceus americanus
- 1 0 0 0 1 0 0 0 1 2 0 0 1 0 0 0 - - -
0 0 1 0 0 0 0 0 ? ? ? - 0 0 1 0 2 0 ? 0
? ? ? ? 0 0 1 0 0 0 0 0 ? 1 - 1 2 0 ? -
- - - 0 0 1 - - - - - - - - - - 0 - - -
- - - 0 0 0 0 0 0 0 ? 1 0 1 2 0 ? 0 0 0
- 0 0 0 ? 0 0 0 0 0 0 0 0 0 0 0 0 0 0 0
0 0 0 0 - ? ? 0 0 0 ? 0 - 3 1 0 0 1 1 0
0 ? ? 0 0 1 1 1 1 ? ? ? 0 - - - - 1 - -
- 0 0 0 - - 0 ? 0 1 - 0 0 1 0 1 1 2 0 0
0 0 0 - 0 0 0 1 4 0

Microdecemplex rolfei
? ? ? ? ? ? 0 0 0 ? ? ? ? ? ? ? ? - - -
0 0 1 0 0 ? ? ? ? ? ? ? 0 0 0 2 ? 0 0
? ? ? ? 0 0 1 0 ? ? 0 0 0 1 ? 1 2 ? ? -
- - - 0 ? 1 - - - - - - - - - ? 0 - - -
- - - 0 0 0 0 0 0 0 ? ? 0 1 1 0 0 0 0 0
0 0 0 0 0 0 0 0 0 0 0 ? 0 ? 0 0 0 0 0 0
0 0 ? 0 - ? 0 0 0 0 ? ? - - - ? 0 ? ? ?
? ? ? ? ? ? ? - - ? ? ? ? ? ? ? ? ? ? ?
? ? ? 0 - - ? ? ? ? ? ? ? ? 0 0 0 0 0 0
0 0 0 - 0 0 0 ? 4 0

Eoarthropleura devonica
? ? ? ? ? ? 0 0 0 ? ? ? ? ? ? ? ? - - -
? ? ? ? ? ? ? ? ? ? ? ? ? 0 0 0 ? ? ? ?

```
?   ?   ?   ?   0   0   ?   ?   ?   ?   ?   ?   ?   ?   ?   ?   ?   ?   ?   ?
?   ?   ?   ?   ?   ?   ?   ?   ?   ?   ?   ?   ?   ?   ?   ?   0   -   -   -
-   -   -   ?   ?   ?   ?   ?   0   0   ?   ?   0   0   1   0   ?   0   0   0
0   0   0   0   0   0   0   0   ?   ?   0   ?   0   ?   0   0   0   0   0   0
0   0   ?   ?   ?   ?   ?   ?   0   0   ?   ?   -   -   -   ?   0   ?   ?   ?
?   ?   ?   ?   ?   ?   0   -   -   ?   ?   ?   ?   ?   ?   ?   ?   ?   ?   ?
?   ?   ?   0   -   -   ?   ?   ?   ?   ?   ?   ?   ?   ?   0   0   0   0   0
0   0   0   -   0   0   1   ?   ?   1
```

Arthropleura sp.

```
?   0   ?   ?   ?   ?   0   0   0   1   2   0   0   0   0   0   ?   -   -   -
1   0   ?   ?   ?   ?   1   ?   ?   ?   ?   -   ?   0   0   0   ?   ?   ?   ?
?   ?   ?   ?   0   0   1   0   0   ?   ?   ?   ?   ?   ?   1   ?   -   ?   ?
?   ?   ?   0   ?   0   1   ?   0   1   0   ?   ?   1   ?   0   0   -   -   -
-   -   -   0   0   0   0   0   0   0   ?   0   0   1   1   0   0   0   0   0
0   0   0   0   0   0   0   0   ?   ?   0   ?   0   ?   0   0   0   0   0   0
0   0   ?   0   -   ?   ?   0   0   0   ?   ?   -   -   -   ?   0   ?   ?   ?
?   ?   ?   0   ?   ?   0   -   -   ?   ?   ?   ?   ?   ?   ?   ?   ?   ?   ?
?   ?   0   0   -   -   ?   ?   ?   ?   ?   ?   ?   ?   ?   0   0   0   0   0
0   0   0   -   0   0   1   ?   5   1
```

Table S9. Morphological character matrix.

Data S1 (separate file)
Phylogeny material. Include the list of morphological characters used for the MP, ML and BI analyses.

Data S2 (separate file)
Morphological characters matrix for the MP analyses.

Data S3 (separate file)
Morphological characters matrix for the BI analyses.

Data S4 (separate file)
Morphological and molecular characters for the total-evidence analyses.

Data S5 (separate file)
Molecular alignment.

Data S6 (separate file)
Partitions of the molecular alignment.

Data S7 (separate file)
Morphological matrix with associated backbone for the constrained analyses

REFERENCES AND NOTES

1. N. S. Davies, R. J. Garwood, W. J. McMahon, J. W. Schneider, A. P. Shillito, The largest arthropod in Earth history: Insights from newly discovered *Arthropleura* remains (Serpukhovian Stainmore Formation, Northumberland, England). *J. Geol. Soc. London* **179**, jgs2021–jgs2115 (2021).

2. J. W. Schneider, R. Werneburg, *Arthropleura*, der größte landlebende Arthropode der Erdgeschichte—Neue Funde und neue Ideen. *Sem. Ther.* **25**, 75–100 (2010).

3. H. M. Wilson, W. A. Shear, Microdecemplicida, a new order of minute arthropleurideans (Arthropoda: Myriapoda) from the Devonian of New York State, USA. *Earth Environ. Sci. Trans. R. Soc. Edinb.* **90**, 351–375 (1999).

4. L. Størmer, Arthropods from the Lower Devonian (Lower Emsian) of Alken-an-der-Mosel, Germany. Part 5: Myriapoda and additional forms, with general remarks on fauna and problems regarding invasion of land by arthropods. *Senckenbergiana Lethaea* **57**, 87–183 (1976).

5. O. Kraus, C. Brauckmann, Fossil giants and surviving dwarfs. Arthropleurida and Pselaphognatha (Atelocerata, Diplopoda): Characters, phylogenetic relationships and construction. *Verdhandlungen des Naturwissenschaftlichen Vereins in Hamburg* **40**, 5–50 (2003).

6. H. M. Wilson, "Palaeobiology of the Arthropleuridea," thesis, The University of Manchester, Manchester, UK (1999).

7. M. Koch, Diplopoda—General morphology, in *Treatise on Zoology - Anatomy, Taxonomy Biology. The Myriapoda* (Brill, 2015), vol. 2, pp. 7–67.

8. O. Kraus, On the structure and biology of *Arthropleura* species (Atelocerata, Diplopoda; upper Carboniferous/lower Permian). *Verhandlungen des naturwissenschaftlichen Vereins Hamburg, Neue Folge* **41**, 5–23 (2005).

9. D. E. G. Briggs, J. E. Almond, The arthropleurids from the Stephanian (Late Carboniferous) of Montceau-les-Mines (Massif Central - France), in *Quand Le Massif Central Était Sous*

l'équateur: Un Écosystème Carbonifère à Montceau-Les-Mines, C. Poplin, D. Heyler, Eds. (Comite des Travaux Historiques et Scientifiques, 1994), pp. 127–135.

10. J. E. Almond, Les Arthropleurides du Stéphanien de Montceau-les-Mines, France. *Bulletin Trimestriel de la Société d'histoire Naturelle et des Amis du Muséum d'Autun* **115**, 59–60 (1985).

11. G. D. Edgecombe, Diplopoda—Fossils, in *Treatise on Zoology - Anatomy*, *Taxonomy*, *Biology. The Myriapoda* (Brill, 2015), vol. 2, pp. 337–352.

12. A. Minelli, *Treatise on Zoology - Anatomy, Taxonomy, Biology. The Myriapoda* (Brill, 2015), vol. 2.

13. R. Fernández, G. D. Edgecombe, G. Giribet, Phylogenomics illuminates the backbone of the Myriapoda Tree of Life and reconciles morphological and molecular phylogenies. *Sci. Rep.* **8**, 83 (2018).

14. L. R. Benavides, G. D. Edgecombe, G. Giribet, Re-evaluating and dating myriapod diversification with phylotranscriptomics under a regime of dense taxon sampling. *Mol. Phylogenet. Evol.* **178**, 107621 (2023).

15. R. S. Sansom, Bias and sensitivity in the placement of fossil taxa resulting from interpretations of missing data. *Syst. Biol.* **64**, 256–266 (2015).

16. W. A. Shear, P. A. Selden, *Eoarthropleura* (Arthropoda, Arthropleurida) from the Silurian of Britain and the Devonian of North America. *Neues Jahrbuch für Geologie und Paläontologie-Abhandlungen* **196**, 347–375 (1995).

17. A. Minelli, *Treatise on Zoology - Anatomy*, *Taxonomy*, *Biology. The Myriapoda* (Brill, 2011) vol. 1.

18. A. Minelli, M. Koch, Chilopoda—General morphology, in *Treatise on Zoology - Anatomy*, *Taxonomy*, *Biology. The Myriapoda* (Brill, 2011), vol. 1, pp. 43–66.

19. S. M. Manton, The evolution of arthropodan locomotory mechanisms. Part 7. Functional requirements and body design in Colobognatha (Diplopoda), together with a comparative account of diplopod burrowing techniques, trunk musculature and segmentation. *Zool. J. Linn. Soc.* **44**, 383–462 (1961).

20. A. Sombke, C. H. G. Müller, The visual system of Myriapoda, in *Distributed Vision: From Simple Sensors to Sophisticated Combination Eyes*, Springer Series in Vision Research, E. Buschbeck, M. Bok, Eds. (Springer International Publishing, 2023), pp. 169–203; https://doi.org/10.1007/978-3-031-23216-9_7.

21. C. H. Müller, A. Sombke, J. Rosenberg, The fine structure of the eyes of some bristly millipedes (Penicillata, Diplopoda): Additional support for the homology of mandibulate ommatidia. *Arthropod Struct. Dev.* **36**, 463–476 (2007).

22. G. D. Edgecombe, C. Strullu-Derrien, T. Góral, A. J. Hetherington, C. Thompson, M. Koch, Aquatic stem group myriapods close a gap between molecular divergence dates and the terrestrial fossil record. *Proc. Natl. Acad. Sci. U.S.A.* **117**, 8966–8972 (2020).

23. W. D. Ian Rolfe, J. K. Ingham, Limb structure, affinity and diet of the Carboniferous 'centipede' *Arthropleura*. *Scottish J. Geol.* **3**, 118–124 (1967).

24. R. B. Davis, N. J. Minter, S. J. Braddy, The neoichnology of terrestrial arthropods. *Palaeogeogr. Palaeoclimatol. Palaeoecol.* **255**, 284–307 (2007).

25. R. G. Netto, C. G. Corrêa, J. H. D. Lima, D. Sedorko, J. Villegas-Martín, Deciphering myriapoda population dynamics during Gondwana deglaciation cycles through neoichnology. *J. South Am. Earth Sci.* **109**, 103247 (2021).

26. S. G. Lucas, A. J. Lerner, J. T. Hannibal, A. P. Hunt, J. W. Schneider, Trackway of a giant *Arthropleura* from the Upper Pennsylvanian of El Cobre Canyon, New Mexico, in *Geology of the Chama Basin* (New Mexico Geological Society, 2005), pp. 279–282; https://nmgs.nmt.edu/publications/guidebooks/details.cfml?ID=118919.

27. J.-D. Moreau, G. Gand, E. Fara, J. Galtier, N. Aubert, S. Fouché, Trackways of *Arthropleura* from the Late Pennsylvanian of Graissessac (Hérault, southern France). *Hist. Biol.* **33**, 996–1007 (2021).

28. J. W. Schneider, S. G. Lucas, R. Werneburg, R. Rößler, Euramerican Late Pennsylvanian/ early Permian arthropleurid/tetrapod associations - implications for the habitat and paleobiology of the largest terrestrial arthropod. *New Mexico Museum of Natural History and Science, Bulletin* **49**, 49–70 (2010).

29. M. A. Whyte, Mating trackways of a fossil giant millipede. *Scottish J. Geol.* **54**, 63–68 (2018).

30. A. Minelli, A. Sombke, Chilopoda—Development, in *Treatise on Zoology - Anatomy*, *Taxonomy*, *Biology. The Myriapoda* (2011), vol. 1, pp. 295–308.

31. A. Minelli, Diplopoda—Development, in *Treatise on Zoology - Anatomy, Taxonomy, Biology. The Myriapoda* (2016), vol. 2, pp. 267–302.

32. V. Perrier, S. Charbonnier, The Montceau-les-Mines Lagerstätte (Late Carboniferous, France). *Comptes Rendus Palevol* **13**, 353–367 (2014).

33. W. T. Calman, III.—On *Arthropleura Moyseyi*, n.sp., from the Coal-Measures of Derbyshire. *Geol. Mag.* **1**, 541–544 (1914).

34. P. Pruvost, La faune continentale du terrain houiller de la Belgique. *Mém. Mus. roy. Hist. Nat Belgique* **44**, 103–282 (1930).

35. J. W. Salter, On some species of *Eurypterus* and allied forms. *Q. J. Geol. Soc. Lond.* **19**, 81–87 (1863).

36. P. Pruvost, *Introduction à l'étude Du Terrain Houiller Du Nord et Du Pas-de-Calais: La Faune Continentale Du Terrain Houiller Du Nord de La France* (Imprimerie Nationale, 1919).

37. P. Pruvost, Les arachnides fossiles du Houiller de Belgique. *Annales de la Société Scientifique de Bruxelles* **41**, 349–355 (1922).

38. P. R. Racheboeuf, J. Vannier, F. R. Schram, D. Chabard, D. Sotty, The euthycarcinoid arthropods from Montceau-les-Mines, France: Functional morphology and affinities. *Earth Environ. Sci. Trans. R. Soc. Edinb.* **99**, 11–25 (2008).

39. M. Lheritier, M. Perroux, J. Vannier, G. Escarguel, T. Wesener, L. Moritz, D. Chabard, J. Adrien, V. Perrier, Fossils from the Montceau-les-Mines Lagerstätte (305 Ma) shed light on the anatomy, ecology and phylogeny of Carboniferous millipedes. *J. Syst. Palaeontol.* **21**, 2169891 (2023).

40. M. Lheritier, G. D. Edgecombe, R. J. Garwood, A. Buisson, A. Gerbe, N. Mongiardino Koch, J. Vannier, G. Escarguel, J. Adrien, V. Fernandez, A. Bergeret-Medina, A. Giupponi, V. Perrier, 3D models related to the publication: Head anatomy and phylogenomics show the Carboniferous giant *Arthropleura* belonged to a millipede-centipede group. *MorphoMuseuM* **e233**, (2024).

41. A. Mirone, E. Brun, E. Gouillart, P. Tafforeau, J. Kieffer, The PyHST2 hybrid distributed code for high speed tomographic reconstruction with iterative reconstruction and a priori knowledge capabilities. *Nucl. Instrum. Methods Phys. Res. B.* **324**, 41–48 (2014).

42. D. Paganin, S. Mayo, T. E. Gureyev, P. R. Miller, S. W. Wilkins, Simultaneous phase and amplitude extraction from a single defocused image of a homogeneous object. *J. Microsc.* **206**, 33–40 (2002).

43. A. Lyckegaard, G. Johnson, P. Tafforeau, Correction of ring artifacts in X-ray tomographic images. *Int. J. Tomo. Stat.* **18**, 1–9 (2011).

44. M. D. Sutton, R. J. Garwood, D. J. Siveter, D. J. Siveter, Spiers and VAXML; A software toolkit for tomographic visualisation, and a format for virtual specimen interchange. *Palaeontol. Electron.* **15**, 15.2.5T (2012).

45. G. D. Edgecombe, G. Giribet, Myriapod phylogeny and the relationships of Chilopoda in *Biodiversidad, Taxonomía y Biogeografia de Artrópodos de México: Hacia Una Síntesis de Su Conocimiento*, J. Llorente Bousquets, J. J. Morrone, Eds. (Prensas de Ciencias, Univ. Nacional Autónoma de México, 2002), vol. 3, pp. 143–168.

46. P. A. Goloboff, S. A. Catalano, TNT version 1.5, including a full implementation of phylogenetic morphometrics. *Cladistics* **32**, 221–238 (2016).

47. F. Ronquist, M. Teslenko, P. van der Mark, D. L. Ayres, A. Darling, S. Höhna, B. Larget, L. Liu, M. A. Suchard, J. P. Huelsenbeck, MrBayes 3.2: Efficient Bayesian phylogenetic inference and model choice across a large model space. *Syst. Biol.* **61**, 539–542 (2012).

48. P. O. Lewis, D. L. Swofford, Back to the future: Bayesian inference arrives in phylogenetics. *Trends Ecol. Evol.* **16**, 600–601 (2001).

49. A. Rambaut, A. J. Drummond, D. Xie, G. Baele, M. A. Suchard, Posterior summarization in Bayesian phylogenetics using Tracer 1.7. *Syst. Biol.* **67**, 901–904 (2018).

50. O. Chernomor, A. Von Haeseler, B. Q. Minh, Terrace aware data structure for phylogenomic inference from supermatrices. *Syst. Biol.* **65**, 997–1008 (2016).

51. B. Q. Minh, H. Schmidt, O. Chernomor, D. Schrempf, M. Woodhams, A. Von Haeseler, R. Lanfear, IQ-TREE 2: New models and efficient methods for phylogenetic inference in the genomic era. *Mol. Biol. Evol.* **37**, 1530–1534 (2020).

52. S. Kalyaanamoorthy, B. Q. Minh, T. K. Wong, A. Von Haeseler, L. S. Jermiin, ModelFinder: Fast model selection for accurate phylogenetic estimates. *Nat. Methods* **14**, 587–589 (2017).

53. D. T. Hoang, O. Chernomor, A. Von Haeseler, B. Q. Minh, L. S. Vinh, UFBoot2: Improving the ultrafast bootstrap approximation. *Mol. Biol. Evol.* **35**, 518–522 (2018).

54. S. Secretan, Les arthropodes du Stéphanien de Montceau-les-Mines. *Bulletin trimestriel de la Société d'Histoire Naturelle et des Amis du Muséum d'Autun* **94**, 23–35 (1980).

55. M. Boule, Sur des débris d' *Arthropleura* trouvés en France. *Bulletin de la Société de l'Industrie Minérale* **3**, 619–638 (1893).

56. K. Andrée, Weiteres über das carbonische Arthrostraken-Genus *Arthropleura* Jordan. *Palaeontographica (1846–1933)* **60**, 295–310 (1913).

57. C. R. Scotese, *Atlas of Permo-Carboniferous Paleogeographic Maps (Mollweide Projection), Maps 53–64, Volumes 4, The Late Paleozoic, PALEOMAP Atlas for ArcGIS, PALEOMAP Project, Evanston, IL* (Digital Commons at Universisty of South Florida, 2014); https://digitalcommons.usf.edu/kip_articles/381.
