# Supplementary material for: Head anatomy and phylogenomics show the Carboniferous giant Arthropleura belonged to a millipede-centipede group
Source: Sci Adv. 2024 Oct 9;10(41):eadp6362. doi: 10.1126/sciadv.adp6362 (PMC11463278; doi:10.1126/sciadv.adp6362)
Supplement: Supplementary file 2 — Data S1 to S7 [file sciadv.adp6362_data_s1_to_s7.zip › adp6362_data_s1.pdf]

# Head anatomy and phylogenomics show the Carboniferous giant *Arthropleura* belonged to millipede-centipede group

by MICKAEL LHERITIER[1,2], GREGORY D. EDGECOMBE[3], RUSSELL J. GARWOOD[3,4], ADRIEN BUISSON[1], ALEXIS GERBE[1], NICOLÁS MONGIARDINO KOCH[5], JEAN VANNIER[1], GILLES ESCARGUEL[2], JEROME ADRIEN[6], VINCENT FERNANDEZ[7], AUDE BERGERET-MEDINA[8], ALEXANDRA GIUPPONI[1] and VINCENT PERRIER[1]

[1]Universite Claude Bernard Lyon1, LGL-TPE, UMR 5276,Villeurbanne, 69100, France.

[2]Universite de Lyon, Université Claude Bernard Lyon 1, CNRS, ENTPE, UMR 5023 LEHNA, F-69622, Villeurbanne, France.

[3]The Natural History Museum, London SW7 5BD, United Kingdom.

[4]Department of Earth and Environmental Sciences, University of Manchester, Manchester M13 9PL, UK.

[5]Scripps Institution of Oceanography, University of California San Diego, La Jolla, CA, USA.

[6]Laboratoire MATEIS. INSA Lyon. Jules Verne building. 21, avenue Jean Capelle. 69621 Villeurbanne Cedex. France.

[7]European Synchrotron Radiation Facility, 71 rue des Martyrs, 38000 Grenoble.

[8]Muséum d'Histoire Naturelle d'Autun. 14, rue Saint Antoine. 71400 Autun. France.

Corresponding author. Email: mickael.lheritier@univ-lyon1.fr

# Supplementary data S1: Phylogeny material

Table 1: List of morphological characters used for the MP, ML and BI analyses. Characters come from Fernández *et al.* (2018). Characters 187-190 were added by the authors.

| # | Description |
|---|---|
| Ontogenetic characters | |
| 1 | Egg tooth on embryonic cuticle of second maxilla: (0) absent; (1) present. |
| 2 | Pattern of segment addition in ontogeny: (0) hemianamorphosis; (1) euanamorphosis; (2) epimorphosis. |
| 3 | Number of fully-formed trunk leg pairs in first post-embryonic/post-pupoid stadium: (0) three; (1) four; (2) forcipule+four; (3) forcipule+12. |
| 4 | Brood care: (0) absent; (1) female (male in *Brachycybe*) bends ventrally around egss; (2) female bends dorsally around eggs. |
| 5 | Peripatoid and foetoid stadia guarded by mother: (0) absent; (1) present. |
| 6 | Pupoid stage: (0) absent; (1) present. |
| 7 | Nauplius larva: (0) absent; (1) present. |
| 8 | Holometaboly: (0) absent; (1) present. |
| Head characters | |
| 9 | Structure of appendage on tritocerebral segment: (0) antenna; (1) chelicera |
| 10 | Variability in number of antennal articles: (0) variable within the species; (1) fixed within the species. |
| 11 | Number of antennal articles: (0) 15 or more; (1) 14; (2) eight or fewer. |
| 12 | Proportions of antennal articles: (0) at least some approximately as long as wide; (1) consistently ring-like, much wider than long. |
| 13 | Distal part of antenna biramous, with three flagella: (0) absent; (1) present. |
| 14 | Antenna with apical cones: (0) apical cones absent; (1) apical cones present. |
| 15 | Schaftorgan on antennal scape: (0) absent; (1) present. |
| 16 | Globulus on distal part of ventral antennal branch: (0) absent; (1) present. |
| 17 | Two lateral areas bearing sensilla basiconica on terminal antennal article: (0) absent; (1) present. |
| 18 | Number of cheliceral segments: (0) three; (1) two. |
| 19 | Plagula ventralis in chelicera: (0) absent; (1) present. |
| 20 | Scorpionid chelate pedipalps: (0) absent; (1) present. |
| 21 | Flattened head capsule: (0) head capsule domed; (1) flattened, with head bent posterior to the clypeus. |

| | |
|---|---|
| 22 | Transverse cephalic suture: (0) absent; (1) present (frontal lie or frontal sulcus); (2) present, divided near lateral margin into antenocellar structure. |
| 23 | Anterior tentorial apodemes: (0) absent; (1) present. |
| 24 | Structure of anterior tentorium: (0) separate, rod-like anterior tentorial arms; (1) an unpaired roof. |
| 25 | Posterior tentorial apodemes: (0) absent; (1) 'present'. |
| 26 | Fenestrated plate composed of fused transverse tendons of mandibular, mx1, and mx2 segments: (0) absent; (1) present. |
| 27 | Structure of lateral eye: (0) stemmata; (1) compound/facetted; (2) single ocellus; (3) absent; (4) simple lens with cup-shaped retina. |
| 28 | Four ocelli in rhomboid cluster: (0) absent; (1) present. |
| 29 | Number of cellular components in eye: (0) facultative constancy; (1) all cellular components highly variable in number. |
| 30 | Circumretinular sheath cells: (0) absent; (1) present. |
| 31 | Interocellar sheath cells: (0) absent; (1) present. |
| 32 | Inverted median eyes: (0) absent; (1) present. |
| 33 | Tömösváry organs: (0) absent; (1) present. |
| 34 | Trichobothria innervated by several sensory cells: (0) absent; (1) present. |
| 35 | Cuticle calcification: (0) absent; (1) present. |
| 36 | Entognathy (overgrowth and mandibles and maxillae by cranial folds): (0) absent; (1) present. |
| 37 | Dentition of labral midpiece / intermediate part: (0) undefined midpiece (median tooth/teeth lacking); (1) single strong tooth; (2) three teeth; (3) several (>3) small teeth. |
| 38 | Single transverse seta projecting medially from labral side piece / lateral part: (0) absent; (1) present. |
| 39 | Labral sidepiece / lateral part incised medially: (0) not incised; (1) incised. |
| 40 | A-shaped epipharyngeal support (labral trapezoid): (0) absent; (1) present. |
| 41 | Arching of border between labral and clypeal part of epipharynx: (0) subtransverse or gently arched distally; (1) strongly arched distally. |
| 42 | Bilobate border between labral and clypeal parts of epipharynx: (0) absent; (1) present. |
| 43 | Single row of bottle-shaped glandular shafts at border between labral and clypeal parts of epipharynx: (0) absent; (1) present. |
| 44 | Single row of bullet-shaped sensilla at proximal margin of field of branching spines at border between labral and clypeal parts of epipharynx: (0) absent; (1) present. |
| 45 | Stomotheca (formed by coxapophyses of palp and leg 1): (0) absent; (1) present. |

46 Tritosternum / sternapophysis adjacent to palpal coxa: (0) absent (coded for post-oral somite II); (1) present.

47 Structure of appendage on post-tritocerebral segment: (0) pedipalp or locomotory leg; (1) mandible.

48 Four sclerites of mandible intersect at cruciform suture: (0) absent; (1) present.

49 Mandible composed of two sclerites (lamina condylifera only sclerite differentiated from flank of mandible): (0) absent; (1) present.

50 Wide membranous band between sclerites of mandible, Haarpolster on a discrete sclerite: (0) absent; (1) present.

51 Arrangement of pectinate lamellae on mandible: (0) multiple imbricated lamellae; (1) lamellae arranged in a single file.

52 Dentate lamellae on mandible: (0) present; (1) absent.

53 Number of groups of teeth in dentate lamella of mandible: (0) three; (1) four/five.

54 Internal and external teeth on mandible: (0) absent; (1) present.

55 Haarpolster a large lobe with dense, uniform setation: (0) absent; (1) present.

56 First maxilla: (0) absent (segmentally homologous appendage as a locomotory limb; (1) present.

57 First maxilla coalesced with sternal intermaxillary plate: (0) absent; (1) present, with unfused stipital and intermaxillary components; (2) mental elements of gnathochilarium consolidated.

58 Gnathochilarial lamellae lingulaes: (0) separate; (1) ’connected’ ("fused").

59 Median suture on first maxillary coxosternite: (0) coxae medially coalesced, separated by median suture; (1) coxae fused, without median suture.

60 Number of articles in telopodite of first maxilla: (0) two; (1) one.

61 "Curled appendages" along inner margin of telopodite of first maxilla: (0) absent; (1) present.

62 Brush-like setae along inner margin of telopodite of first maxilla: (0) absent; (1) with short, curved barbs; (2) plumose, branching as slender hairs.

63 Plumose setae on coxal process of first maxilla: (0) absent; (1) present.

64 Maxillary organ: (0) absent; (1) present.

65 Maxillary nephridia: (0) paired; (1) fused; (2) absent.

66 Limbless postmaxillary segment: (0) limbs present; (1) absent.

67 Coxae of second maxilla: (0) coxae separate; (1) coxae fused.

68 Metameric pores on second maxillary coxosternum: (0) minute opening of secondary maxillary gland medial to mx2 coxosternite; (1) enlarged opening of mx2 gland (metameric pore")incorporated in medial part of mx2 coxosternite; (2) metameric pore on lateral part of mx2 coxosternite.

| | |
|---|---|
| 69 | Form of second maxillary telopodite: (0) slender, leg-like, with elongate prefemur/femur; (1) short, stout. |
| 70 | Trochanter on second maxilla: (0) present; (1) absent. |
| 71 | Pair of spine bristles at distal end of tibia of second maxilla: (0) absent; (1) present. |
| 72 | Plumose setae on inner surface of tarsus of second maxillary telopodite: (0) absent (simple setae); (1) plumose setae present. |
| 73 | Comb-like fringe of setae on distal article of telopodite of second maxilla: (0) absent; (1) bifurcating of multifurcating spines; (2) densely-aligned simple bristles. |
| 74 | Termination of telopodite of second maxilla: (0) simple (no claw or setae); (1) claw; (2) seta or setigerous tubercle. |
| 75 | Structure of claw of telopodite of second maxilla: (0) unipartite, conical claw; (1) thick, elongate digits with interspersed thin digits; (2) hook-like claw. |
| 76 | Postmaxillary sclerites: (0) absent; (1) present. |
| Body characters | |
| 77 | Forcipule with fang and venom gland: (0) absent; (1) present. |
| 78 | Pleurite of forcipular segment arching over coxosternite: (0) absent (small pleurite); (1) pleurite arching over coxosternum, discontinuous medially; (2) pleurite arching over coxosternum, continuous ventromedially. |
| 79 | Forcipular tooth plate: (0) absent; (1) anteriorly projecting serrate endite; (2) transverse sclerotied band on anterior margin. |
| 80 | Porodont on forcipular coxosternite: (0) absent; (1) transluent, seta-like porodont. |
| 81 | Forcipular coxosternite sclerotised in midline: (0) coxae separated medially, with sternite present in adult; (1) coxosternal plates meeting medially, with flexible hinge; (2) coxosternal plates meeting medially, hinge sclerotised and non-functional. |
| 82 | Forcipular coxosternite deeply embedded into cuticle above second trunk segment: (0) not embedded; (1) embedded. |
| 83 | Coxalplatten (forcipular coxosternal apodemes): (0) absent; (1) present. |
| 84 | Tarsungulum on forcipular/first trunk segment: (0) separate tarsus and pretarsus; (1) tarsus and pretarsus fused |
| 85 | Spine comb on tarsus of forcipule/first trunk leg: (0) absent; (1) present. |
| 86 | Basal node on forcipular tarsus/tarsungulum: (0) absent; (1) present. |
| 87 | Hinge between articles of forcipular telopodite: (0) between prefemur/trochanteroprefemur and femur; (1) between trochanteroprefemur and tibia; (2) between trochanteroprefemur and tarsungulum. |
| 88 | Spine bristle on inner edge of forcipular trochanteroprefemur opposed to four spine bristles on each coxal margin: (0) absent; (1) present. |

89 Tergite of forcipular segment: (0) separate tergite; (1) separate tergite lacking, fused to next posterior segment.

90 Limb VII as chilaria: (0) absent; (1) present.

91 Number of post-forcipular leg-bearing segments (in Chilopoda): (0) 15; (1) 21; (2) 47-51.

92 Intraspecific variability in number of leg pairs: (0) constant number of leg pairs; (1) variable number of leg pairs.

93 Hexapod thoracic-abdominal tagmosis: (0) absent; (1) present.

94 Body segments fused into diplosegments: (0) absent; (1) present.

95 Fusion of tergites, pleurites and sternites: (0) absent (free pleurites and sternites); (1) pleurotergites (pleurae fused to tergites, with free sternites); (2) complete body rings (tergites, pleurites and sternites fused).

96 "Special heterotergy" (alternating long and short tergites, with reversal of lengths between seventh and eighth walking leg-bearing segments): (0) absent; (1) present.

97 Overlap between tergite 1 and head shield: (0) head shield overlpas tergite 1; (1) tergite 1 overlaps head shield.

98 Second tergite much larger than the following ones: (0) absent; (1) present.

99 Meso- and metathorax in mature stages bearing wings: (0) absent; (1) present.

100 Single large tergal plate over trunk segments 7-9: (0) separate tergites; (1) single tergite.

101 Long tergites divided into two: (0) absent; (1) present.

102 Intercalary sclerites: (0) absent or weakly sclerotised; (1) small intercalary tergites (pretergites) and sternites; (2) strongly developed intercalary tergites and sternites.

103 Paramedian sutures or grooves on tergum (Längsnähten): (0) absent; (1) present.

104 Crescentic sulci on most trunk tergites: (0) absent; (1) present.

105 Tergite margination: (0) absent or on last tergite only; (1) on most or all tergites.

106 Tergite projections: (0) absent or on last tergite only; (1) present on at least TT11 and 13.

107 Large unpaired tergal spines associated with spine bristles, aligned longitudinally on midline: (0) absent; (1) present.

108 Tergal spicula (hairs): (0) absent; (1) present.

109 Paired sternal paramedian pores with valves: (0) absent; (1) present.

110 Sternal pore areas/sternal glands: (0) absent in at least females; (1) present in both sexes.

111 Endosternite: (0) absent; (1) present.

112 Coxal vesicles: (0) absent; (1) present at limb base on numerous trunk segments; (2) on distal part of firstabdominal segment as ventral tube.

| | |
|---|---|
| 113 | Leg pentagonal in cross-section, with marginal spines on the angles: (0) absent; (1) present. |
| 114 | Proliferation of telopodal glands on posterior legs: (0) absent; (1) present. |
| 115 | Socketed spurs D/V, a/m/p on distal extremities of podomeres: (0) absent; (1) present. |
| 116 | Bipartite division of tarsi of anterior series of trunk legs: (0) absent; (1) present. |
| 117 | Tarsi divided into many joints: (0) tarsi undivided or bisegmented; (1) tarsus flagelliform, with many joints. |
| 118 | Tarsal spurs: (0) absent; (1) present. |
| 119 | Tarsus 1 with pair of terminal spurs: (0) absent; (1) present. |
| 120 | Tarsal papillae and resilient sole hairs: (0) absent; (1) present. |
| 121 | Slit sensilla: (0) absent; (1) present. |
| 122 | Claspers: (0) absent; (1) present. |
| 123 | Coxal pouches on legs: (0) absent; (1) present. |
| 124 | Ultimate leg-bearing segment a complete cylinder: (0) absent; (1) present. |
| 125 | Coxopleurites on ultimate legs: (0) coxa and pleurites fused as short coxopleurite; (1) elongate coxopleurite. |
| 126 | Ultimate leg thickened in males: (0) inconspicuous sexual dimorphism with respect to proportions of ultimate leg; (1) present. |
| 127 | Ultimate leg trochanter: (0) present; (1) minute or absent |
| 128 | Trochanter of ultimate and penultimate legs with ventral spine: (0) absent; (1) present |
| 129 | Furcula: (0) absent; (1) present |
| 130 | Aculeus: (0) absent; (1) present |
| 131 | Antenna and leg regeneration: (0) present; (1) absent |
| 132 | Relationship of sternal and lateral longitudinal muscles: (0) united sternal and lateral longitudinal muscles; (1) separate sternal and lateral longitudinal muscles, with separate segmental tendons. |
| 133 | Anisostigmophory: (0) absent (spiracles present on all trunk segments from second pedigerous segment); (1) present (spiracles associated with long tergites only). |
| 134 | Shape of stigmatic plates: (0) flat, tirangular; (1) divided in midline; (2) sterna nodifera sensu Blanke & Wesener (2014) (character 20); (3) sliding sternites sensu Blanke & Wesener (2014) (character 20). |
| 135 | First and second stigmatic plate: (0) with tracheae; (1) without tracheae, used for muscles attachment. |
| 136 | Longitudinal and transverse connections between segmental tracheal branches: (0) absent; (1) present. |
| 137 | Chiasmata: (0) absent; (1) present. |

| | |
|---|---|
| 138 | Arrangement of tracheal pouches in comparison to body axis: (0) diagonal orientation; (1) longitudinal orientation. |
| 139 | Apodemes of mid-body tracheal pouches: (0) absent; (1) present, serving as muscles attachment. |
| 140 | Branching of mid-body tracheal pouch apodemes: (0) uniramous; (1) biramous. |
| 141 | Foregut with differentiated gizzard with plicate walls: (0) absent; (1) present. |
| 142 | Posterior part of foregut organised as a sieve with stiff, anteriorly directed projections: (0) absent; (1) present. |
| 143 | Midgut developed within the yolk: (0) midgut cells enclose entire yolk; (1) lumen of embryonic midgut lacking yolk globules. |
| 144 | Intestine shape: (0) straight tube; (1) N-shaped, bent twice. |
| 145 | Malpighian tubules formed as endodermal extensions of midgut: (0) absent; (1) present. |
| 146 | Malpighian tubules formed as ectodermal extensions of hindgut: (0) absent; (1) present. |
| 147 | Lateral defense glands: (0) absent; (1) present. |
| 148 | Shape of defence glands: (0) elongate subtubular; (1) subspherical. |
| 149 | Defence secretions with benzoquinines: (0) absent; (1) present. |
| Sexual characters | |
| 150 | Testes differentiated into macrotestis with ampulla and microtestis: (0) present; (1) absent. |
| 151 | Lateral testicular vesicles linked by a central, posteriorly extended deferens duct: (0) absent; (1) present. |
| 152 | Testicular vesicles spindle shaped: (0) absent; (1) present. |
| 153 | Female gonopod on first genital segment: (0) absent; (1) present. |
| 154 | Female gonopod used to manipulate single eggs: (0) absent; (1) present. |
| 155 | Female gonopod segmentation: (0) three articles and claw, with basal articles of gonopod pair separated; (1) two articles, the proximal article of each gonopod pair partly joined (syntelopodite), the distal article a spine; (2) two articles, the proximal article of each gonopod pair partly joined (syntelopodite), the distal article a spine. |
| 156 | Female gonopod with basal article bearing spurs (macrosetae) and terminal article with a broad claw: (0) absent; (1) present. |
| 157 | Claw of female gonopod fused with the apical article: (0) claw separate; (1) claw fused. |
| 158 | Position of male gonopore: (0) opisthogoneate; (1) behind coxa of second pair of trunk legs; (2) through coxa of second pair of trunk legs; (3) on fourth trunk segment. |

| | |
|---|---|
| 159 | Segmentation of male gonopod on first genital segment: (0) two segments; (1) single segment, rudimentary; (2) unsegmentded style. |
| 160 | Male gonopod on second genital segment: (0) present; (1) absent. |
| 161 | Form of male gonopod on second genital segment: (0) blunt cones; (1) slender styles. |
| 162 | Bivalved anogenital capsule: (0) absent; (1) present. |
| 163 | Anal organs: (0) absent; (1) present through ontogeny; (2) present only in juveniles. |
| 164 | Coxal organs: (0) absent; (1) present. |
| 165 | Serial distribution of coxal organs: (0) on last four pairs of legs; (1) on last pair of legs only. |
| 166 | Arrangement of coxal pores: (0) few pores in linear row; (1) numerous small pores scattered over coxopleure or large pore field; (2) opening in depressions between meshwork of ridges. |
| 167 | Spermatophore web produced by Spinngriffel on first genital segment of male: (0) absent; (1) present. |
| 168 | Bean-shaped spermatophore with tough, multi-layered wall: (0) absent; (1) present |
| 169 | Sperm dimorphism: (0) absent; (1) microsperm and macrosperm present |
| 170 | Sperm flagellum: (0) present; (1) absent. |
| 171 | Coiling of sperm flagellum around nucleus: (0) absent (filiform); (1) present. |
| 172 | Spiral ridge on nucleus of sperm: (0) absent; (1) present. |
| 173 | Sperm nucleus with machette of microtubules: (0) absent; (1) present. |
| 174 | Pseudoperforatorium: (0) absent; (1) present. |
| 175 | Spermathecae formed by paired pockets in mouth cavity: (0) absent; (1) present. |
| 176 | Modified leg(s) in male on seventh and/or eight trunk segment(s): (0) absent; (1) present. |
| 177 | Leg 8 in adult males: (0) walking leg (unmodified); (1) accessory gonopod; (2) functional gonopod. |
| 178 | Leg 9 in adult males: (0) walking leg (unmodified); (1) accessory gonopod; (2) functional gonopod. |
| 179 | Leg 10 in adult males: (0) walking leg (unmodified); (1) functionnal gonopod. |
| 180 | Posterior male leg pair as telopods with inner horns: (0) absent; (1) present. |
| Other characters | |
| 181 | Pre-anal segment with trichobothria bearing a long sensory seta: (0) absent; (1) present. |
| 182 | Appendage on opisthosomal segment I: (0) present; (1) absent. |
| 183 | Lamellate respiratory organs derived from posterior wall of trunk limb buds: (0) absent; (1) present. |

| | |
|---|---|
| 184 | Type of lamellate opisthosomal respiratory organs: (0) book gills; (1) book lungs. |
| 185 | Thoracic food groove (invagination of sternites) and filter-feeding apparatus: (0) absent; (1) present. |
| 186 | Serrate setae arranged in lateral and caudal tufts: (0) absent; (1) present. |
| 187 | Ventral B- and K-plates at base of limbs: (0) absent; (1) present. |
| 188 | Mandibular comb lamellae: (0) absent; (1) present. |
| 189 | Number of podomeres in locomotory legs: (0) 3; (1) 4; (2) 5; (3) 6; (4) 7; (5) 8; (6) 10 and more; (7) variable. |
| 190 | Subdivision of the tergites: (0) not subdivided; (1) subdivided between a central syntergite and lateral paratergites. |

# References

Blanke, Alexander, & Wesener, Thomas. 2014. Revival of forgotten characters and modern imaging techniques help to produce a robust phylogeny of the Diplopoda (Arthropoda, Myriapoda). *Arthropod structure & development*, **43**(1), 63–75.

Fernández, Rosa, Edgecombe, Gregory D, & Giribet, Gonzalo. 2018. Phylogenomics illuminates the backbone of the Myriapoda Tree of Life and reconciles morphological and molecular phylogenies. *Scientific Reports*, **8**(1), 1–7.
